# The link between macrophage polarization and response to radiotherapy in cancers: mechanisms and therapeutic opportunities

**DOI:** 10.3389/fimmu.2026.1767192

**Published:** 2026-06-03

**Authors:** Zibing Zhao, Zhuangzhuang Zheng

**Affiliations:** 1 The First Clinical College of Jilin University, Changchun, Jilin, China; 2 Department of Radiation Oncology, The First Hospital of Jilin University, Changchun, China

**Keywords:** M1 macrophages, M2 macrophages, macrophage polarization, radioresistance, radiotherapy, tumor microenvironment

## Abstract

Macrophages, as central players in the tumor microenvironment (TME), exhibit remarkable plasticity, shifting between pro-inflammatory M1 and immunosuppressive M2 states. This polarization directly influences the response to radiotherapy in cancers. While M1 macrophages promote antitumor immunity, M2 macrophages contribute to immune evasion, metastasis, and treatment resistance. Ionizing radiation, while designed to kill tumor cells, can inadvertently alter macrophage polarization within the TME. High-dose and particle-based radiotherapies tend to favor M1 polarization, enhancing tumor control, while conventional radiotherapy often induces M2 macrophages, promoting radioresistance. These shifts in macrophage phenotype are driven by changes in metabolic signaling, hypoxia, and cytokine production within the TME, which together dictate the outcome of therapy. Emerging strategies aim to manipulate macrophage polarization to overcome radiotherapy-induced resistance, including the use of immune checkpoint inhibitors, nanoparticles, and metabolic reprogramming agents. By targeting macrophage recruitment, survival, and reprogramming, these therapies can potentially improve the efficacy of radiotherapy and reduce tumor recurrence. Understanding and leveraging macrophage plasticity holds promise for optimizing cancer treatment and enhancing patient outcomes in the era of precision oncology.

## Graphical abstract

**Figure:**
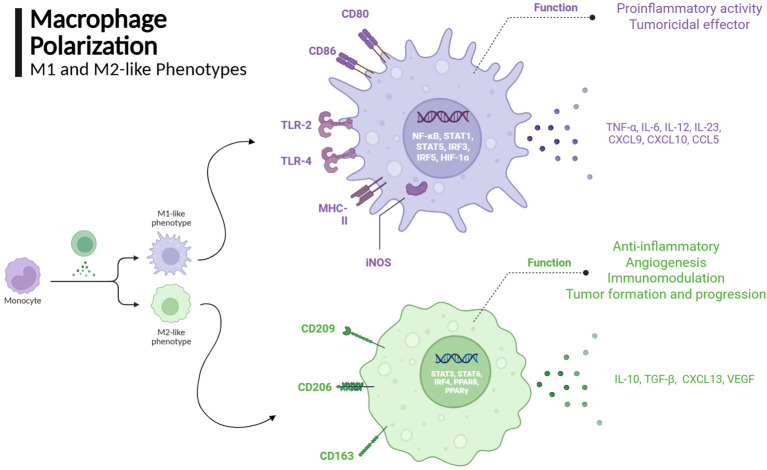

## Highlights

- Tumor-associated macrophages (TAMs) exhibit plasticity, influencing radiotherapy outcomes.
- Ionizing radiation triggers TAM polarization toward immunosuppressive M2-like phenotypes, enhancing radioresistance.
- Strategies targeting TAM reprogramming, such as CSF-1R inhibitors and CD^40^ agonists, can improve radiotherapy efficacy.
- Metabolic and extracellular vesicle-mediated pathways modulate macrophage polarization post-radiotherapy.
- Emerging radiotherapy modalities modulate macrophage phenotype to enhance antitumor immunity.

## 1 Introduction

Radiotherapy remains a cornerstone of curative treatment for solid malignancies, utilized in over 50% of cancer patients to induce DNA damage and cell death. Despite its widespread efficacy, intrinsic and acquired radioresistance frequently leads to locoregional failure and disease recurrence, significantly limiting long-term survival rates. Contemporary oncology has shifted focus beyond cancer cell-intrinsic factors to the tumor microenvironment (TME), which actively orchestrates therapeutic response and resistance. Within this complex ecosystem, infiltrating immune cells dictate the balance between tumor eradication and tissue repair, with myeloid lineage cells often dominating the landscape (1–3).

**Figure 1:**
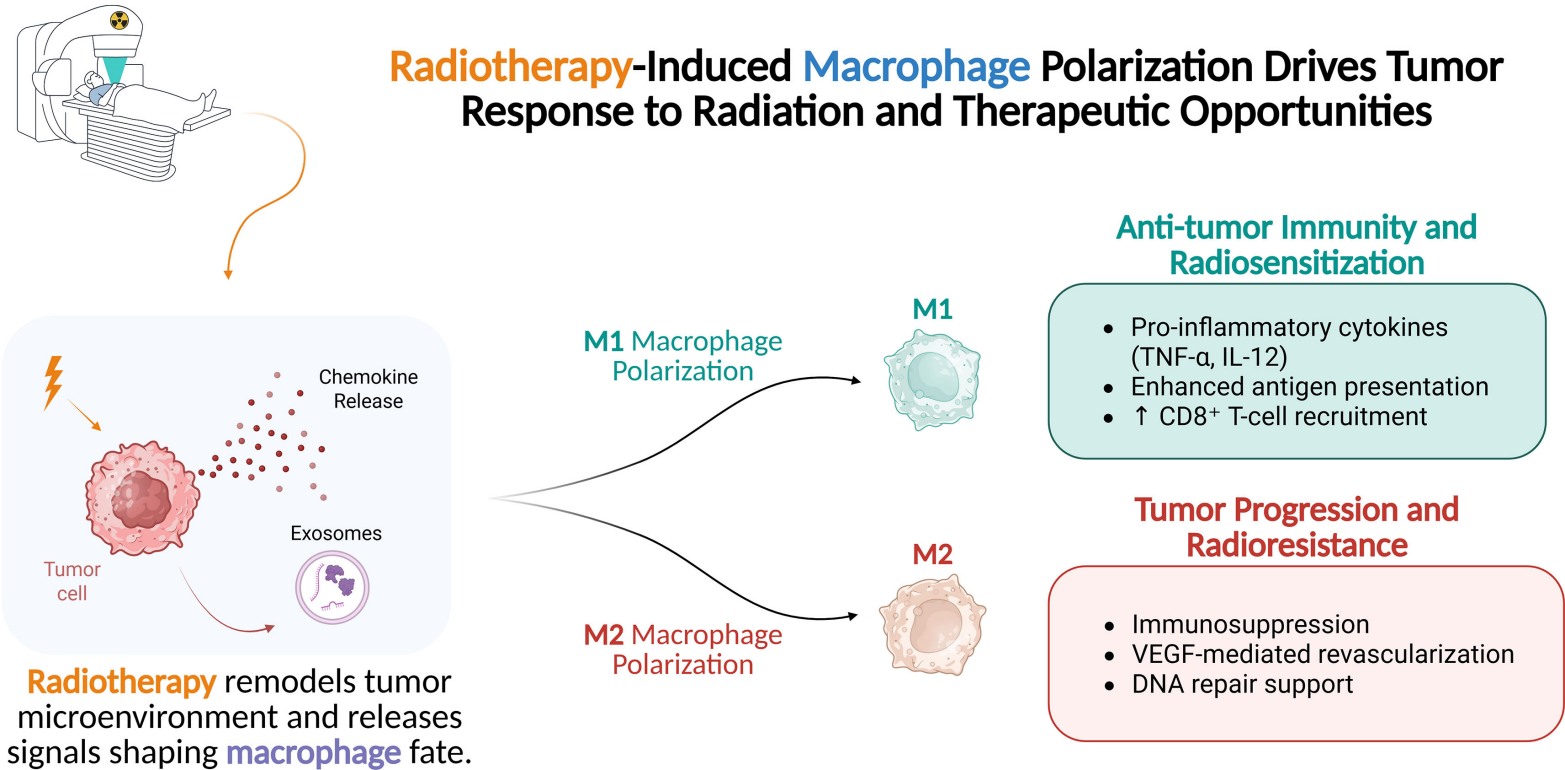
Differentiation of macrophages into pro-inflammatory-like (M1) (left) and anti-inflammatory-like (M2) (right) phenotypes. M1 macrophages, marked by molecules such as CD^80^, CD^86^, and iNOS, are involved in promoting inflammation and tumoricidal activity. In contrast, M2 macrophages, characterized by CD^163^, CD^206^, and CD^209^, play roles in anti-inflammatory responses, angiogenesis, immunomodulation, and tumor progression. The polarization is influenced by various signaling pathways and transcription factors.

Tumor-associated macrophages (TAMs) represent the most abundant immune population in the TME, originating from either tissue-resident progenitors or recruited bone marrow-derived monocytes. Unlike fixed-phenotype immune cells, macrophages possess plasticity, enabling them to continuously reprogram their functional state in response to microenvironmental signals, a dynamic process termed polarization. This adaptive capacity allows macrophages to serve as either frontline defenders against malignancy or as unwitting collaborators in tumor progression, depending on the dominant biochemical cues in their immediate vicinity (4).​ At the extremes of this polarization spectrum lie two archetypal states with opposing functions. Classically activated M1 macrophages (see **Figure 1**) arise in response to interferon-gamma (IFN-γ), lipopolysaccharide (LPS), and granulocyte-macrophage colony-stimulating factor (GM-CSF), which activate transcription factors such as nuclear factor kappa-light-chain-enhancer of activated B cells (NF-κB) and drive expression of major histocompatibility complex class II (MHC-II) molecules. These cells function as tumoricidal effectors, secreting pro-inflammatory cytokines including tumor necrosis factor (TNF), IL-12, and IL-6, while directly phagocytosing malignant cells and activating cytotoxic CD^8+^ T cells and natural killer (NK) cells. Metabolically, M1 macrophages rely predominantly on aerobic glycolysis and produce reactive oxygen species (ROS) and nitric oxide (NO) through upregulated inducible nitric oxide synthase (iNOS), which mediate direct cytotoxicity (5, 6).

Conversely, alternatively activated M2 macrophages (see **Figure 1**) differentiate in response to anti-inflammatory cytokines such as IL-4, IL-10, and IL-13, activating signaling pathways including mTORC2, HIF-2α, AMPK, and PPARs. These pathways redirect cellular metabolism toward oxidative phosphorylation fueled by fatty acid oxidation and glutaminolysis, enabling sustained tissue remodeling activities. M2 macrophages express high levels of arginase-1 (Arg-1), mannose receptor (CD^206^), and scavenger receptors, and secrete immunosuppressive factors like transforming growth factor-beta (TGF-β) and IL-10 alongside pro-angiogenic mediators such as vascular endothelial growth factor (VEGF). While these functions are essential for wound healing and tissue homeostasis in normal physiology, cancer cells exploit M2 TAMs to facilitate angiogenesis, extracellular matrix remodeling, immune evasion, and metastatic dissemination (6, 7). Importantly, the M1/M2 dichotomy represents an oversimplified model, as TAMs *in vivo* display a continuum of intermediate and mixed phenotypes shaped by the complex, heterogeneous TME. Further M2 subtypes have been characterized, including M2a (induced by IL-4/IL-13, promoting Th2 responses), M2b (induced by immune complexes, involved in immune regulation), M2c (induced by IL-10, facilitating tissue remodeling), and M2d (involved in angiogenesis). During early-stage tumorigenesis, the macrophage population often favors an M1 phenotype, exerting antitumor pressure; however, as malignancies progress and the TME becomes increasingly hypoxic, acidic, and enriched with tumor-derived factors such as lactate, succinate, and tumor-derived exosomes, the balance shifts decisively toward immunosuppressive M2-like states. This shift is further reinforced by tumor-secreted metabolites that modulate gene expression and reprogram macrophage signaling pathways, ultimately granting TAMs robust tumor-supportive capacities (6).

Although the M1/M2 framework is widely used as a convenient shorthand, it represents two ends of a multidimensional activation spectrum rather than discrete or stable macrophage lineages. In tumors, macrophages frequently exhibit mixed, transitional, or time-evolving programs, and single-cell or spatial profiling has revealed multiple TAM states that do not map cleanly onto M1 versus M2 categories (8).

Radiotherapy parameters themselves are upstream determinants of macrophage state. Clinically, tumors are treated using heterogeneous regimens like conventional fractionation (~1.8 to 2 Gy per fraction), hypofractionation (typically ~3 to 8 Gy per fraction), stereotactic ablative RT/SBRT (often ≥8 to 20 Gy per fraction), and emerging approaches such as ultra-high dose-rate (FLASH) delivery, each producing distinct patterns of cell death, damage-associated molecular patterns (DAMPs) release, vascular injury or normalization, and inflammatory cytokine kinetics that shape TAM recruitment and polarization. In parallel, radiation quality (low-linear energy transfer (LET) photons vs intermediate-LET protons vs high-LET heavy ions such as carbon) alters DNA damage complexity, redox stress, and innate immune sensing, with downstream consequences for whether TAMs adopt inflammatory/antigen-presenting programs versus wound-healing, fibrotic, and immunosuppressive states. Accordingly, throughout this review we treat macrophage “polarization” after radiotherapy as the net result of regimen-dependent tumor or stromal cues and direct myeloid reprogramming, rather than a single uniform RT effect (9–12).

Paradoxically, while ionizing radiation aims to destroy the tumor, the resulting cellular injury triggers a *wound healing* response that recruits innate immune cells to the irradiated site. Radiation-induced tissue damage upregulates chemokines such as CCL2 and CSF-1, which actively attract circulating monocytes into the tumor bed. Once infiltrated, the hypoxic and inflammatory conditions created by radiation can bias infiltrating macrophages toward M2-like programs rather than M1-like programs, although mixed and time-dependent trajectories are common. This phenomenon creates a negative feedback loop where the treatment itself fortifies the stromal support system of the surviving cancer cells. Importantly, macrophages are not only shaped indirectly by irradiated tumor or stromal signals; they are also directly exposed to ionizing radiation during treatment. Direct irradiation can trigger macrophage-intrinsic DNA damage responses, redox and innate immune signaling (NF-κB and type I interferon programs), resulting in dose-dependent shifts across inflammatory, reparative, and antigen-presenting states (5, 13).

An increase in M2-like TAM programs after radiotherapy has been associated with poor prognosis and treatment failure across multiple solid tumor types. These cells confer radioresistance through diverse mechanisms, including the scavenging of radiation-induced reactive oxygen species (ROS) and the secretion of growth factors like VEGF that promote revascularization. Furthermore, M2 macrophages suppress the adaptive immune response by inhibiting T-cell infiltration and activity, thereby preventing the “abscopal effect” necessary for systemic disease control. Consequently, the TME shifts toward an immunosuppressive, pro-fibrotic state that protects residual tumor cells from subsequent fractionated doses (4, 14).

Disrupting the crosstalk between irradiated tumor cells and macrophages presents a promising avenue to overcome radioresistance and enhance therapeutic ratios. Current strategies include depleting TAM populations using CSF-1R inhibitors or inhibiting their recruitment by blocking the CCL2-CCR2 axis. More elegantly, reprogramming approaches aim to shift tumor-promoting M2-like TAM programs toward more inflammatory, antigen-presenting (M1-like) states using agents such as CD^40^ agonists or PI3Kγ inhibitors.

Direct macrophage irradiation occurs during clinical radiotherapy and can independently reshape phenotype, even in the absence of tumor-cell–derived cues. Human monocyte-derived macrophages remain viable after clinically relevant fractionated irradiation and exhibit increased expression of antigen-presenting or pro-inflammatory markers with NF-κB activation, supporting the concept that radiotherapy can “prime” macrophages toward more inflammatory and immune-interacting states under some conditions (15).

Dose and scheduling strongly influence the direction of polarization along a spectrum rather than a strict M1/M2 switch. *In vivo* and ex vivo evidence indicates that certain low-dose regimens can program macrophages toward an iNOS^+^/M1-like, T-cell–permissive state that improves immune trafficking and tumor control, whereas other contexts (including post-irradiation wound-repair milieus) promote mixed or reparative programs that can support regrowth (16, 17).

Mechanistically, direct irradiation activates interconnected macrophage-intrinsic pathways: (i) DNA damage response and stress signaling (ATM/ATR–p53 and downstream transcriptional changes), (ii) redox signaling (ROS-driven modulation of NF-κB and MAPKs), and (iii) innate nucleic-acid sensing triggered by cytosolic DNA species and micronuclei, engaging cGAS–STING-IRF3/NF-κB-type I interferon outputs. These programs can enhance antigen presentation, inflammatory chemokines, and cross-talk with T cells, but can also contribute to chronic tissue-injury phenotypes depending on dose, tissue context, and macrophage ontogeny (18, 19).

Radiotherapy shapes TAM state via two routes; (1) indirect re-education by irradiated tumor, stromal, or vascular signals and extracellular vesicles, and (2) direct macrophage irradiation that reprograms transcriptional, metabolic, and innate immune pathways. The net macrophage phenotype observed in tumors therefore reflects the balance of these routes plus local metabolites (hypoxia or lactate), cytokines, microbiome-derived signals, and therapy combinations.

This review explores the mechanistic link between macrophage polarization and radiotherapy response through the lens of treatment parameters, then maps how these variables reshape tumor or stromal signaling, myeloid-intrinsic programs, and combination strategies to improve clinical outcomes.

## 2 Radiation-induced plasticity: molecular and metabolic reprogramming

Throughout the following sections, we interpret macrophage responses after radiotherapy as dynamic state transitions shaped by dose and fractionation and microenvironmental context, and we avoid over-interpreting static M1/M2 labels when evidence supports mixed or transient phenotypes.

Although the M1/M2 framework is widely used as a convenient shorthand, it represents two ends of a multidimensional activation spectrum rather than discrete or stable macrophage lineages. In tumors, macrophages frequently exhibit mixed, transitional, and time-evolving programs, and the phenotypes observed after radiotherapy may not map cleanly onto binary M1 versus M2 categories. Although more granular subsets such as M2a, M2b, M2c, and M2d have been described, most radiotherapy studies do not provide sufficient phenotypic resolution to distinguish these populations systematically, particularly in human tumors. In addition, commonly used markers such as CD68, CD80, CD86, CD163, CD206, iNOS, and Arg-1 do not always correspond to discrete functional states when interpreted in isolation. Therefore, throughout this review, we use the terms “M1-like” and “M2-like” as functional shorthand rather than rigid classifications, while recognizing that radiotherapy often induces hybrid and context-dependent macrophage states. This limitation is also therapeutically relevant, because strategies aimed at a simple “M2-to-M1 switch” may not fully capture the complexity of macrophage reprogramming in human tumor microenvironments.

### 2.1 Metabolic adaptations and hypoxic signaling

Radiation-induced macrophage plasticity is closely shaped by metabolic rewiring in both macrophages and irradiated tumor cells, as well as by hypoxia-associated signaling within the tumor microenvironment. Across several preclinical models, these pathways influence whether macrophages adopt a pro-inflammatory, M1-like phenotype or an immunosuppressive, M2-like state, thereby modifying the overall response to radiotherapy.

One line of evidence comes from metabolic changes within the macrophage compartment itself. In an autochthonous medulloblastoma model, Ni et al. found that proton ultrahigh-dose-rate FLASH radiotherapy, compared with standard-dose-rate irradiation after a single 10-Gy fraction, shifted intratumoral macrophages toward an M1-like phenotype, with increased CD^80^ and CD^86^ and reduced CD^206^ and arginase-1. This effect was accompanied by lower reactive oxygen species (ROS), reduced oxidized LDL, and suppression of peroxisome proliferator-activated receptor gamma (PPARγ) activity, suggesting that in this setting radiotherapy disrupted a ROS–lipid peroxidation–PPARγ axis that would otherwise favor immunosuppressive macrophage programming. Importantly, these findings indicate that the effect of ROS after radiotherapy is context dependent rather than uniformly pro-tumorigenic, since oxidative stress may either support immunosuppressive remodeling or contribute to inflammatory antitumor responses depending on the pathway involved (20). A related metabolic mechanism was described in glioma, where Lu et al. identified glutamine synthetase as a mediator of radiation-induced M2 polarization. Radiotherapy increased signal transducer and activator of transcription 5 (STAT5) signaling and glutamate-ammonia ligase expression in glioma-associated macrophages, whereas pharmacologic inhibition with L-methionine sulfoximine reversed this shift, restored M1-associated markers, reduced glioma invasion *in vitro*, and improved tumor control *in vivo* when combined with radiotherapy. This combination was also associated with reduced angiogenesis and greater CD^8+^ T-cell infiltration, linking amino acid metabolism to both macrophage polarization and treatment efficacy (21).

A second group of studies highlights the role of tumor-derived metabolic signals in shaping macrophage behavior after irradiation. In head and neck squamous cell carcinoma, Meng et al. showed that radiotherapy altered tumor NAD+ metabolism and increased the release of niacinamide-rich extracellular vesicles (EVs). These vesicles suppressed macrophage NF-κB signaling through USP7 binding, promoted an M2-skewed phenotype, and reduced IL-6 and IL-8 secretion. Inhibition of nicotinamide phosphoribosyl transferase (NAMPT), the key NAD+ biosynthetic enzyme, lowered niacinamide levels in tumor-derived EVs, restored macrophage NF-κB activity, increased M1-like polarization, enhanced lymphocyte infiltration, and sensitized tumors to radiotherapy (22). In hepatocellular carcinoma, Liu et al. similarly found that radiotherapy, although capable of recruiting CD8+ T cells, simultaneously promoted M2 polarization through tumor-derived metabolic remodeling. This effect was linked to HIF-1α-driven glycolysis and lactate production. The natural compound Liensinine counteracted this pathway by activating AMP-activated protein kinase (AMPK) and promoting HIF-1α degradation, thereby reducing lactate secretion, favoring M1 polarization, and improving the antitumor activity of radiotherapy, particularly when combined with anti-PD-L1 therapy (23).

Hypoxia-related signaling provides an additional layer of regulation. In glioblastoma, Rosberg et al. showed that hypoxic stress increased C3 expression and generated C3a, which acted through C3a receptor on macrophages to promote an M2-like phenotype in perivascular niches. Pharmacologic blockade of C3aR did not reduce total macrophage numbers but selectively decreased the M2 fraction. When combined with radiotherapy, C3aR inhibition prolonged survival and reduced tumor vascularity in murine glioma models, indicating that hypoxia-driven complement signaling can function as a barrier to effective radiation response (24).

Taken together, these studies show that radiotherapy interacts with metabolic and hypoxic pathways at multiple levels to shape macrophage polarization. ROS-linked lipid signaling, glutamine metabolism, NAD+/niacinamide-dependent EV communication, lactate production, and hypoxia-associated complement activation can each tilt the balance between M1-like and M2-like macrophage states. As a result, the metabolic context of irradiation helps determine whether macrophages support immune activation and treatment response or instead reinforce an immunosuppressive, radioresistant microenvironment.

### 2.2 The role of extracellular vesicles and intercellular communication

**Figure 2:**
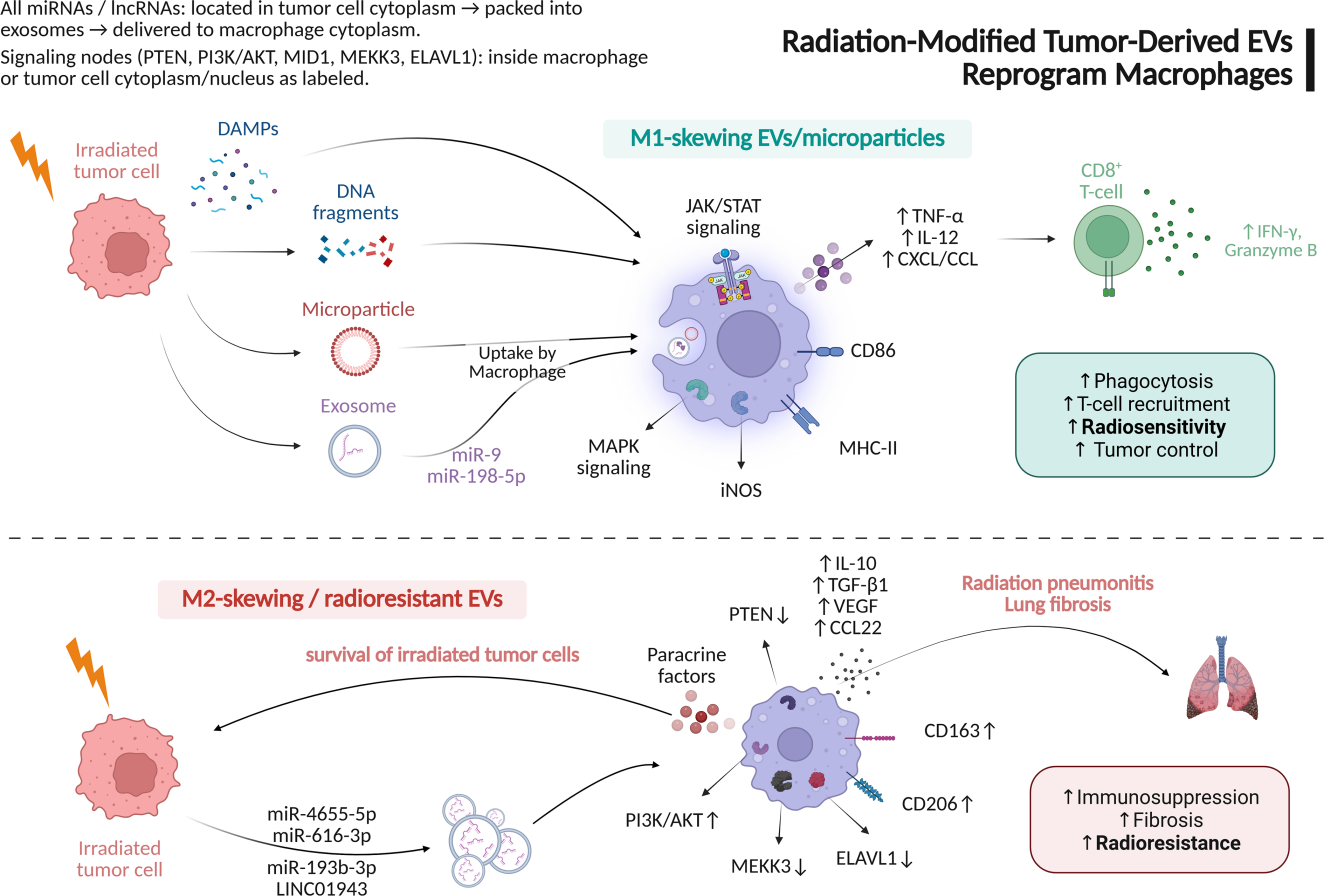
Role of radiation-modified tumor-derived exosomes and microparticles in reprogramming macrophages, leading to shifts toward M1-like or M2-like macrophage programs. Irradiated tumor cells release exosomes and microparticles containing miRNAs and other signaling factors that influence macrophage behavior. M1-skewing EVs enhance antitumor immunity, increasing phagocytosis and T-cell recruitment, while M2-skewing EVs promote immunosuppression, fibrosis, and radioresistance, contributing to tumor survival and potential radiation-induced lung fibrosis.

EVs, including exosomes and larger microparticles, are increasingly recognized as important mediators of communication between irradiated tumor cells and macrophages. By transferring miRNAs, proteins, and damage-associated signals, EVs can reshape macrophage phenotype after radiotherapy. Notably, this effect is context dependent. In some models and patient cohorts, radiation-associated EVs promote macrophage repolarization toward an M1-like, antitumor state and enhance immune activation. In others, EVs derived from irradiated, metastatic, or radioresistant tumor cells drive macrophages toward an M2-like, immunosuppressive phenotype that supports tumor persistence, fibrosis, or radioresistance. EV-mediated signaling is also bidirectional, as macrophage-derived vesicles can in turn alter tumor cell radiosensitivity (**Figure 2**).

Among the clearest examples of EV-mediated antitumor reprogramming are studies of radiotherapy-induced tumor cell–released microparticles (RT-MPs). In malignant pleural effusion and melanoma models, RT-MPs acted as bystander mediators that induced ferroptotic, immunogenic cell death, increased calreticulin exposure and ATP release, and enhanced macrophage phagocytosis. These vesicles were preferentially taken up by TAMs, where they promoted a shift from an M2-like to an M1-like phenotype, reduced CD^206^ expression, increased phagocytic activity, and improved survival. When combined with programmed cell death protein-1 (PD-1) blockade, RT-MPs produced stronger tumor control and durable CD^8+^ T-cell memory, and depletion studies showed that both macrophages and CD^8+^ T cells were required for therapeutic benefit, linking RT-MP-induced macrophage reprogramming to both local tumor control and systemic antitumor immunity (25). A related study in prostate cancer further strengthened this concept by loading RT-MPs with RSL3 and CT20p (RC@RMPs). These engineered vesicles enhanced oxidative and iron stress in macrophages, increased CD^86^ expression more strongly than unloaded RT-MPs, reduced intratumoral M2 macrophages, and improved tumor control, particularly in combination with anti-PD-1 therapy. Again, macrophage and CD8+ T-cell depletion attenuated the antitumor effect, underscoring the immunologic dependence of this strategy (26). Similar findings were reported in a murine model of non-small cell lung cancer (NSCLC) brain metastasis, where RT-MPs crossed the blood-brain barrier, accumulated in metastatic lesions, and were preferentially internalized by TAMs and microglia. Compared with microparticles from non-irradiated cells, RT-MPs increased IL-12 and iNOS, reduced Arg1, expanded M1-like TAM subclusters, strengthened chemokine signaling to T cells, reduced metastatic burden, and prolonged survival, with additional benefit from anti-PD-1 therapy (27).

Evidence from human tumors also supports a role for radiation-associated EVs in promoting M1-like macrophage polarization. In rectal cancer, low-dose irradiation of ex vivo tumor explants shifted TAMs from an M2-dominant baseline toward an M1-like phenotype, accompanied by enhanced phagocytosis and reduced PD-1 expression. Resected tumors from patients treated with neoadjuvant short-course radiotherapy similarly showed a higher intratumoral M1/M2 ratio than non-irradiated lesions. In organotypic colorectal cancer models, EVs isolated from irradiated DLD-1 cells were sufficient to reproduce this M2-to-M1 transition, suggesting that vesicle cargo can directly transmit the immunologic effects of radiation (28). In cervical cancer, Ren et al. reported that post-radiotherapy tumor biopsies contained more TAMs with a more M1-like profile than pretreatment samples. Plasma EVs collected after radiotherapy, but not before treatment, induced M2 macrophages to adopt a more M1-like phenotype with reduced PD-1 expression and enhanced phagocytic function, indicating that radiotherapy alters EV content in a functionally meaningful way (29). Likewise, in oral squamous cell carcinoma, irradiated tumor cells released EVs enriched in miR-198-5p, which were taken up by macrophages and promoted M1-like polarization, with increased CD^80^, CD^86^, and IL-12 and reduced CD^163^ and CD^206^. Mechanistically, miR-198-5p directly targeted MYD88, and MYD88 overexpression reversed the EV-induced phenotypic shift, supporting a specific miRNA-dependent pathway for radiation-mediated macrophage reprogramming (30). In human papillomavirus (HPV) positive head and neck squamous cell carcinoma, exosomes enriched in miR-9 similarly promoted M1 polarization by suppressing peroxisome proliferator–activated receptor delta (PPARδ) in macrophages. These macrophages then enhanced radiation-induced DNA damage in tumor cells, as reflected by increased phosphorylated H2A histone family member X (γH2AX) foci, while patient data linked higher miR-9 and iNOS expression with improved radiotherapy response and survival (31).

However, the effects of radiation-associated EVs are not uniformly beneficial. In several tumor types, EVs released after irradiation instead promote M2-like macrophage polarization and an immunosuppressive microenvironment. In NSCLC, irradiation increased exosome release and enriched vesicles in miR-4655-5p. Transfer of these exosomes to macrophages induced cytokine changes consistent with an M2-associated, anti-inflammatory, and fibrogenic phenotype. Mechanistically, miR-4655-5p targeted Midline-1 E3 ubiquitin ligase (MID1) and altered the MID1/PP2Ac axis, which was linked to M2-skewed polarization (32). A second NSCLC study identified a similar effect for exosomal miR-616-3p. Irradiation increased miR-616-3p in tumor-derived exosomes, and these vesicles promoted M2 polarization in recipient macrophages through phosphatase and tensin homolog (PTEN) suppression and PI3K/AKT activation. Patient samples further supported this association, as radiotherapy was accompanied by increased miR-616-3p, CD163, and CD^206^ expression and reduced PTEN levels (33). In triple−negative breast cancer (TNBC), radiation-induced exosomes enriched in LINC01943 drove M2-associated macrophage features by inhibiting ELAVL1-mediated autophagy, thereby supporting tumor progression (34). Similarly, in nasopharyngeal carcinoma, exosomes from highly metastatic cells delivered miR-193b-3p to macrophages, repressed MEKK3, and induced a tumor-promoting macrophage phenotype whose secretory products enhanced tumor radioresistance and clonogenic survival (35).

Importantly, EV-mediated communication is not limited to tumor-to-macrophage signaling. In endometrial cancer, M2-polarized macrophages released exosomes containing hsa_circ_0001610, which acted in tumor cells as a sponge for miR-139-5p. This increased Cyclin B1 expression, weakened radiation-induced G2/M arrest, and ultimately promoted radioresistance, illustrating how macrophage-derived vesicles can reinforce therapeutic failure from the opposite direction (36).

These studies show that EVs are central mediators of intercellular communication after irradiation, but their functional consequences depend on tumor type, vesicle origin, and molecular cargo. In rectal and cervical cancer, as well as in several experimental models using RT-MPs or miRNA-enriched exosomes, radiation-associated vesicles favor M1-like polarization, greater phagocytic activity, and improved antitumor immunity (25–31). In contrast, in NSCLC, triple-negative breast cancer, and nasopharyngeal carcinoma, radiation-conditioned or metastasis-associated vesicles drive M2-like polarization and support radioresistance or tissue injury (32–35). Thus, EV signaling represents a major mechanism by which radiotherapy can either reinforce immune-mediated tumor control or promote an immunosuppressive, treatment-resistant microenvironment.

### 2.3 Genomic, epigenetic, and regulatory drivers of polarization

Radiotherapy engages a range of genomic, transcriptional, and epigenetic programs in tumor cells and myeloid populations that converge on macrophage recruitment and polarization. For conceptual clarity, true epigenetic mechanisms in this context refer primarily to chromatin-level regulation, including histone modification, chromatin-reader activity, and, more broadly, DNA methylation-dependent control of gene expression. By contrast, non-coding RNAs, kinase signaling, DNA damage sensors, and cytokine or growth factor networks should be viewed as broader regulatory mechanisms that can interact with epigenetic states but are not themselves direct epigenetic modifications. Across the included models, these radiation-induced molecular changes are directly linked to shifts toward either immunostimulatory M1-like or immunosuppressive M2-like phenotypes, with corresponding effects on radiosensitivity and systemic responses.

In esophageal squamous cell carcinoma, Nakajima and colleagues demonstrated that radiotherapy reshapes the microenvironment through tumor cell-intrinsic cyclic GMP–AMP synthase (cGAS)-stimulator of interferon genes (STING) signaling. While oligofractionated radiation generated cytosolic double-stranded DNA (dsDNA) and activated type I interferon signaling to recruit cytotoxic T cells, it simultaneously triggered an immunosuppressive response by upregulating IL-34 mRNA in a cGAS-STING-dependent manner. This upregulation, which was suppressed by ATR/ATM blockade, correlated with the accumulation of CD^163^ M2 TAMs in patient specimens, suggesting that radiation-driven innate immune sensing drives a dual phenotype characterized by both T-cell inflammation and M2-TAM enrichment (37).

Downstream of DNA damage sensing, specific repair proteins further modulate the immune landscape. In hepatocellular carcinoma, ionizing radiation induced dsDNA breaks that activated DNA-dependent protein kinase catalytic subunit, which subsequently phosphorylated and stabilized transforming acidic coiled-coil–containing protein 3. This stabilized TACC3 not only enhanced non-homologous end joining (NHEJ) repair but also increased the expression and secretion of IL-4 and IL-10. Consequently, conditioned media from TACC3-overexpressing cells drove macrophages toward a CD^206^ M2 phenotype, a process reversed by the pharmacological inhibition of TACC3, which sensitized tumors to radiotherapy and increased CD^8+^ T-cell infiltration (38).

Post-transcriptional regulation via non-coding RNAs also plays a critical role in mediating radioresistance and polarization. Although these mediators are often discussed alongside epigenetic regulation because they alter gene-expression programs, they are more accurately classified as post-transcriptional regulatory mechanisms rather than direct epigenetic modifications. Choi et al. identified that radioresistant colorectal cancer cells upregulate miR-1226-5p, which directly suppresses the transcription factor interferon regulatory factor 1. The resulting loss of IRF1 in macrophages, combined with signal transducer and activator of transcription 6 (STAT6) activation, promoted an M2-like program characterized by TGF-β secretion. This macrophage-derived TGF-β, in turn, enhanced epithelial-mesenchymal transition and radioresistance in tumor cells. The circular RNA circSLC43A1 was found to sponge miR-1226-5p, thereby restoring IRF1 expression and preventing the export of the miRNA to macrophages, ultimately mitigating the M2-driven resistance phenotype (39).

Among the mechanisms discussed here, the clearest example of a direct epigenetic regulator is the chromatin reader bromodomain-containing protein 4 (BRD4). In a TNBC model, Kim et al. showed that inhibiting BRD4 during fractionated radiotherapy reduced the infiltration of CD68+ TAMs and shifted the population from CD206+ M2-like cells toward a CD206− M1-like profile. This repolarization was accompanied by downregulated HIF-1α and PD-L1, leading to improved local tumor control and abscopal responses (40). In contrast, other pathways identified in this section are better understood as transcriptional or signaling regulators rather than direct epigenetic mechanisms.

In contrast, Tang et al. identified a signaling-dependent transcriptional program in hepatocellular carcinoma in which maternal embryonic leucine zipper kinase (MELK) activated STAT3 to drive CCL2 expression. This MELK/STAT3/CCL2 axis promoted TAM recruitment and M2 polarization; combining MELK knockdown with radiotherapy suppressed M2 markers, enhanced M1 markers, and amplified antitumor efficacy (41).

Beyond chromatin and gene-expression control, radiotherapy also alters macrophage polarization through broader intercellular regulatory networks, including cytokine secretion, growth-factor signaling, damage-associated molecular patterns, and metabolic stress pathways. Lee et al. found that M2-like TAMs in hepatocellular carcinoma secrete C-X-C motif chemokine ligand 6 (CXCL6), which activates C-X-C motif chemokine receptor 2 (CXCR2) on tumor cells to upregulate DNA repair proteins (including γ-H2AX, Rad50, and p-Chk2) and EMT markers via NF-κB signaling. This CXCL6/CXCR2 axis directly supported the repair of radiation-induced DNA damage, and its blockade sensitized hepatocellular carcinoma cells to radiation (42).

Growth factor signaling induced by radiation also modulates macrophage identity. Im et al. observed that radiotherapy elevated fibroblast growth factor 2 (FGF2) protein levels in colorectal tumors, while TAMs uniquely expressed high levels of fibroblast growth factor receptor 1 (FGFR1) and FGFR2. This radiotherapy-induced FGF2 signaling prevented the acquisition of an M1 phenotype; conversely, blocking FGF2 shifted TAMs toward an iNOS+ inflammatory state and improved the efficacy of fractionated radiotherapy (43).

In contrast to pro-tumorigenic shifts, radiation can also trigger immunogenic signaling through damage-associated molecular patterns. Zhu et al. demonstrated that irradiated breast cancer cells release high mobility group box 1 (HMGB1), which activates toll-like receptor 4 (TLR-4) on macrophages. This interaction reprogrammed macrophages toward an M1-like, TNF-α-producing phenotype. The resulting TNF-α secretion was essential for suppressing metastatic signaling in non-irradiated cells, thereby mediating the radiation-induced abscopal effect (44).

Finally, metabolic gene signatures link oxidative stress responses to macrophage polarization and ferroptosis. In esophageal squamous cell carcinoma, Xue et al. described an NRF2–GCLM–GPX4 antioxidant axis that protects tumor cells from radiation-induced ferroptosis. High expression of these genes correlated with increased M2 macrophage infiltration, which further promoted radioresistance via acyl-CoA synthetase long-chain family member 4 (ACSL4)-mediated EMT, although this state paradoxically sensitized cells to ferroptosis inducers (45).

In a gastric cancer model, carbon-ion radiotherapy (CIRT) was shown to enhance antitumor immunity by downregulating the enzyme dihydroorotate dehydrogenase (DHODH). Suppression of DHODH triggered ferroptosis and the release of factors that polarized macrophages toward an M1-like state. Overexpression of DHODH prevented this repolarization and reduced the therapeutic efficacy of CIRT, establishing a link between tumor metabolic gene expression, ferroptotic cell death, and macrophage modulation (46).

Collectively, these studies show that radiotherapy reshapes macrophage polarization through a layered network of mechanisms that includes genomic stress responses, authentic epigenetic regulation, post-transcriptional control by non-coding RNAs, transcription factor activation, and broader cytokine, growth-factor, and metabolic signaling. In the revised framework, BRD4-related chromatin regulation represents the clearest epigenetic example discussed in this section, whereas non-coding RNAs, kinase signaling, and secreted mediators are more accurately interpreted as complementary regulatory processes. Together, these pathways influence the secretion of factors such as IL-34, IL-4, CCL2, FGF2, and CXCL6, thereby shaping macrophage phenotype, radioresistance, and tumor survival after irradiation.

### 2.4 Impact of radiation modality: photons, protons, and heavy ions

Macrophage polarization after radiotherapy is influenced not only by dose but also by radiation modality and delivery rate. Across preclinical studies, ablative photon doses, proton therapy, carbon-ion radiotherapy, and ultra-high dose-rate irradiation have each shown the capacity to shift macrophages toward a more pro-inflammatory phenotype, although the magnitude and mechanism of this effect vary by platform.

Among photon-based approaches, dose intensity appears to be a key determinant. In the models examined, ablative radiotherapy delivered at 16 Gy promoted a clear shift toward an M1-like macrophage phenotype, whereas lower doses and conventional fractionation did not produce comparable polarization changes. When combined with anti-PD-L1 therapy, this ablative regimen also enhanced CD8+ T-cell infiltration and generated systemic antitumor effects, suggesting that high-dose photon irradiation may improve checkpoint blockade in part through macrophage reprogramming (47).

Particle therapy may further modify the immune landscape in ways that differ from conventional photons. In studies of proton irradiation, TAMs were repolarized from an M2-dominant state toward a mixed M1/M2 phenotype through NF-κB signaling, particularly via nuclear translocation of the p65 subunit. Pharmacologic inhibition of NF-κB reversed this effect, supporting a mechanistic role for this pathway. Notably, proton-irradiated M1-like macrophages also showed greater radioresistance, associated with improved ROS handling and a sustained DNA damage response marked by increased γH2AX and 53BP1, indicating that proton therapy can alter both macrophage phenotype and stress adaptation (9).

Distinct immunologic effects have also been reported with heavier ions. In pancreatic ductal adenocarcinoma models, CIRT produced a stronger antitumor immune profile than photon irradiation. Whereas photon therapy generated a mixed response that included fibrosis and immunosuppressive features, CIRT was associated with more prominent M1 macrophage signatures and greater immune checkpoint activation in genetically engineered mouse models. These findings suggest that carbon ions may create a microenvironment more favorable for combination with immune checkpoint inhibitors than standard photon therapy in this setting (48).

Emerging delivery strategies such as ultra-high dose-rate radiotherapy (UHDR-RT) may similarly influence macrophage behavior. In head and neck squamous cell carcinoma models, UHDR-RT delivered at dose rates of at least 40 Gy per second shifted macrophages from an M2-like to an M1-like phenotype more effectively than conventional dose-rate irradiation. This change was accompanied by stronger CD8+ T-cell activation, increased tumor cell apoptosis, impaired DNA damage repair, and abscopal immune effects in non-irradiated tumors, supporting the idea that delivery rate, in addition to dose and particle type, can shape the immunologic consequences of radiotherapy (49).

These studies indicate that the physical characteristics of radiotherapy are important determinants of macrophage plasticity. High-dose photons, proton irradiation, carbon ions, and ultra-high dose-rate delivery all appear capable of shifting macrophages toward a more inflammatory state, although by different mechanisms and in different tumor contexts. These modality-specific effects may partly explain differences in synergy with immunotherapy and should be considered when evaluating how radiation can be used to remodel the tumor microenvironment.

## 3 The pro-tumorigenic shift: drivers of resistance and recurrence

### 3.1 Mechanisms of radioresistance and immunosuppression

Focusing on radioresistance, a study by Zhang et al. on colorectal cancer highlights the significance of eukaryotic translation initiation factor 5A (EIF5A), a protein associated with radioresistance. The exploration demonstrated that EIF5A upregulation was linked to the presence of cancer stem cells (CSCs) and a suppressive tumor immune microenvironment, characterized by reduced infiltration of CD^8+^ T cells and M1 macrophages, alongside an increase in M2 macrophages and regulatory T cells. By knocking down EIF5A, the radioresistance was reversed and enhanced tumor control, emphasizing EIF5A as a potential therapeutic target to reshape the tumor immune microenvironment and boost immune responses during radiotherapy (50).

In TNBC, Lee et al. investigated the impact of radiotherapy on macrophage polarization, particularly the shift toward M2 macrophages. They found that radiotherapy induced an M2 macrophage phenotype, which in turn contributed to tumor recurrence by promoting local tumor invasion. M2 macrophages secreted IL-6, a cytokine that enhanced tumor cell invasiveness. Neutralizing IL-6 significantly reduced tumor cell infiltration into the irradiated stroma, suggesting that targeting macrophage-mediated pathways could reduce relapse and improve radiotherapy outcomes in TNBC (51).

The involvement of TAMs in radioresistance was further explored in a study by Yang et al. on glioma and high-dose stereotactic radiosurgery (SRS). In an intracranial glioma mouse model, single-fraction SRS was delivered at 20 Gy or a dose-escalated 40 Gy, the latter representing the high-dose condition. The authors observed that in tumors responding to high-dose SRS, there was an increase in M1 macrophage polarization, which correlated with improved tumor control. In contrast, a higher presence of M2 macrophages was associated with poor therapeutic outcomes and tumor recurrence. The findings suggest that macrophage polarization, particularly the M1/M2 ratio, could be an important predictor of response to SRS, and that strategies to manipulate TAM polarization might improve the response to radiotherapy in glioblastoma treatment (52).

Wang et al. also identified hematopoietic stem and progenitor cells (HSPCs) as critical contributors to tumor regrowth after radiotherapy. The study found that radiation therapy promoted HSPC differentiation into M2 macrophages in the TME, a process driven by increased CSF-1 expression. Blocking this pathway with the CSF-1 inhibitor GW2580 prevented HSPC differentiation into M2 macrophages, significantly enhancing tumor control and survival. This study provides a novel mechanism of tumor regrowth post-radiation and suggests that targeting the HSPC-to-M2 macrophage pathway could improve cancer therapy outcomes (53).

Although M1-skewed macrophage states are generally associated with improved tumor control in many experimental models, this relationship is not universally preserved in clinical cohorts, where marker-based macrophage classification may capture more complex inflammatory states. In glioma, Xia et al. reported that higher infiltration of macrophages classified as M1-like was associated with poorer survival in patients receiving radiotherapy, particularly in glioblastoma. This observation should be interpreted cautiously. Rather than indicating that all M1 macrophages are intrinsically detrimental, the finding more likely reflects the limitations of the binary M1/M2 framework in glioma and the possibility that inflammatory macrophage markers may coexist with radioresistant, highly stressed, or treatment-refractory tumor states. Accordingly, in this setting, M1-like infiltration may function more as a prognostic correlate of an adverse microenvironment than as evidence of a uniformly protective macrophage program (54).

Overall, the studies collectively highlight the central role of macrophage polarization in the radioresistance mechanisms of various cancers. In particular, the shift toward M2 macrophages, whether driven by specific signaling molecules or hematopoietic progenitors, often contributes to a more immunosuppressive TME, facilitating tumor recurrence and metastasis. Conversely, M1-like phenotypes are frequently associated with better therapeutic responses in preclinical and translational models, particularly when accompanied by enhanced antigen presentation, cytotoxic T-cell recruitment, and suppression of M2-associated programs. However, their prognostic significance is not uniform across all tumor types. In some clinical settings, especially glioma, macrophages labeled as M1-like may instead reflect a mixed inflammatory state associated with tissue damage, radioresistance, or aggressive disease biology. These observations reinforce that macrophage phenotype should be interpreted in context rather than treated as a universally favorable or unfavorable marker.

### 3.2 Vascular modulation and stromal remodeling

Macrophages contribute to radiotherapy response not only through direct immune functions but also by reshaping the vascular and stromal components of the tumor microenvironment. After irradiation, changes in endothelial signaling, chemokine gradients, and extracellular matrix composition can alter macrophage recruitment and polarization, with important consequences for tumor control, metastasis, and recurrence.

In some settings, radiotherapy can support macrophage-mediated tumor clearance. In glioma models, irradiation induced immunogenic cell death and promoted macrophage reprogramming toward a more inflammatory, tumor-clearing state. This effect was strengthened by combining radiotherapy with anti-CD^47^ monoclonal antibody treatment, which enhanced macrophage phagocytosis and further shifted macrophages toward an effector phenotype through transcriptional and metabolic reprogramming. The combination reduced tumor growth and prolonged survival, illustrating how macrophage plasticity can be exploited to improve radiotherapy efficacy (55).

In contrast, radiotherapy can also remodel the tumor stroma in ways that favor immunosuppression and progression. In breast cancer, post-irradiation extracellular matrix remodeling increased collagen deposition and tissue stiffness, changes that promoted M2-like macrophage polarization. This macrophage state was associated with higher integrin β3 expression and enhanced tumor cell proliferation, suggesting a reciprocal interaction in which stromal remodeling and macrophage polarization reinforce one another to support tumor growth, metastasis, and recurrence (56). A related pro-metastatic pattern has been described in bladder cancer, where irradiated tumor cells secreted CCL2 and recruited CCR2+ myeloid cells into the tumor microenvironment. These recruited macrophages then shifted from an M1-like to an M2-like phenotype, promoting metastasis. Blocking the CCL2-CCR2 axis prevented this polarization change and reduced metastatic spread, indicating that radiotherapy-induced chemokine signaling can indirectly drive tumor progression through macrophage recruitment and reprogramming (57).

Dose may also influence whether vascular and stromal remodeling becomes tumor-promoting or tumor-restraining. Low-dose radiation, typically in the range of 0.5 to 2 Gy per fraction, has been reported to favor M1-like macrophage polarization, with increased iNOS activity and nitric oxide production. In parallel, it can normalize tumor vasculature, reduce proangiogenic signaling pathways such as VEGF and S1P, and improve T-cell infiltration. Together, these changes may convert the microenvironment to a state that is more permissive to immune-mediated tumor rejection and more responsive to therapy (58).

In summary, macrophages play a crucial role in tumor progression and response to radiotherapy through their polarization and interaction with the TME. While radiotherapy can induce a pro-inflammatory, M1-like phenotype that promotes tumor clearance, it may also inadvertently foster a pro-tumor environment by promoting M2 polarization, which enhances metastasis and tumor recurrence. Therefore, targeting macrophage polarization could serve as a therapeutic strategy to enhance the efficacy of radiotherapy and reduce the risk of tumor progression and recurrence.

## 4 Pharmacological and immunological synergy: overcoming resistance

### 4.1 Enhancing checkpoint blockade and CAR-T efficacy

The integration of radiotherapy with immune checkpoint inhibition (ICI) relies heavily on the plasticity of the myeloid compartment to bridge local cytotoxicity with systemic immunity. Study findings are summarized in **Table 1**.

**Table 1:** Combination of radiotherapy and immune-modulating therapies across various cancer models.

Cancer model	RT dose	Combinations	Key macrophage polarization findings	Tumor response	Mechanism	Ref.
Colorectal Cancer (MC38, CT26)	2 x0.5Gy, 2x2Gy	Anti-PD-L1	Increased M1-like (pro-inflammatory) TAMs; M2-like (Arg1-high) decreased in secondary tumors	Improved tumor control, survival, and abscopal effect	TAM polarization to M1 enhances antitumor immunity; JAK-STAT pathway involved	(59)
Lung Carcinoma (LLC), Melanoma (B16-F10), Colon Cancer (MC38)	8Gy × 3	Anti-PD-1, anti-TIGIT	M1 TAMs increased in primary and abscopal tumors, M2 decreased	Significant tumor suppression and survival improvement	M1 TAMs activate CD8+ T cells via TNF-α, CXCL10, CCL5; macrophage depletion abolishes effect	(60)
Hepatocellular Carcinoma (HCC)	3x6 Gy	Anti-PD-1 + VEGFR2 blockade	M1 TAMs increased in non-irradiated tumors with anti-VEGFR2, M2 decreased	Enhanced local and abscopal tumor control, lung metastasis reduction	VEGFR2 shifts TAMs to M1, enhancing CD8+ T cell recruitment and tumor response	(61)
Triple-negative Breast Cancer (4T1)	24 Gy (3x8 Gy)	PI3Kγδ inhibitor (duvelisib) + Anti-PD-1	M2 TAMs decreased, M1 TAMs increased with PI3Kγδ inhibition	Strongest tumor suppression with triple therapy, abscopal effect	PI3Kγδ inhibition reverses RT-induced M2 polarization, enhances T cell activity	(62)
NSCLC (344SQR), Pancreatic Cancer (PANC-02)	12 Gy × 3	SHP-2 inhibition + Anti-PD-L1	M1 TAMs increased, M2 TAMs decreased with triple therapy	Improved tumor control, survival, abscopal effect	SHP-2 inhibition shifts TAMs from M2 to M1, promotes CD8+ T cell function	(63)
Triple-negative Breast Cancer (MDA-MB-231, 4T1)	8 Gy × 2	CDK4/6 inhibitor (Abemaciclib) + Anti-PD-1	M1 TAMs increased, M2 TAMs decreased in RT + Abemaciclib + anti-PD-L1	Maximal tumor growth inhibition with triple therapy	Enhanced IFN-γ production, improved T cell infiltration, M1 repolarization	(64)
Oral Squamous Cell Carcinoma (MOC2), Lewis Lung Carcinoma (LLC)	6 Gy × 1	Radiosensitizer (Fe3O4@MS-NH2) + TLR7 agonist + CTLA-4 blockade	M1 TAMs increased, M2 TAMs and MDSCs decreased	Strong local and abscopal tumor suppression, systemic immune activation	Radiosensitizer and immune modulation increase ROS and cytokine production, enhance tumor immunity	(65)
Pancreatic Ductal Adenocarcinoma (Panc02)	8 Gy single dose	CLDN18.2-targeted CAR-T therapy	M1 TAMs increased, M2 TAMs decreased with RT + CAR-T	Enhanced tumor control, prolonged survival	RT induces M1 polarization, increases CAR-T infiltration and effector function	(66)
High-grade Glioma (CT-2A)	4 Gy	RT + Temozolomide + Anti-PD-1	M2 TAMs decreased, total TAMs and MDSCs reduced	Improved survival with RT + TMZ, no significant PD-1 blockade effect	RT reduces suppressive myeloid populations, enhances T-cell infiltration	(67)

RT, radiotherapy.

In murine models of colorectal cancer, low-dose radiation (0.5–2 Gy per fraction, delivered in two fractions) combined with PD-L1 blockade effectively polarized TAMs toward an M1-like phenotype. This pro-inflammatory shift was essential for the observed abscopal effect, as macrophage depletion in secondary, unirradiated tumors completely abolished the systemic immune response, suggesting that M1-polarized macrophages are necessary effectors for synergy of radiation and ICI in metastatic settings (59).

Similar mechanistic dependencies were observed when combining radiotherapy with dual PD-1 and T-cell immunoreceptor with Ig and ITIM domains (TIGIT) blockade. In this context, the combination therapy drove M1 macrophage polarization characterized by elevated expression of nitric oxide synthase 2 (Nos2) and MHC-I/II within primary tumors and, to a lesser degree, in abscopal lesions. These M1 macrophages were functionally required to enhance CD^8+^ T-cell activation and reduce exhaustion markers; their depletion eliminated both local control and the establishment of durable immune memory (60).

To further amplify these systemic effects, targeting specific vascular and chemokine pathways has proven effective. In hepatocellular carcinoma models, radiotherapy combined with anti-PD-1 and anti-VEGF receptor 2 (VEGFR2) therapy shifted TAMs toward an M1 phenotype via STAT1-dependent signaling. These repolarized macrophages secreted high levels of C-C motif chemokine ligand 5 (CCL5), which acted as a critical recruiter for tumor-specific CD^8+^ T cells trafficking to distant, unirradiated sites, thereby enabling the abscopal response (61).

However, radiation-induced remodeling often requires pharmacological assistance to prevent compensatory immunosuppression. In TNBC models, radiation alone induced an increase in M2-polarized TAMs and immunosuppressive signaling. The addition of a PI3Kγδ inhibitor (duvelisib) to radiation and PD-1 blockade reversed this trend, reducing M2 markers and promoting M1 differentiation. This triple combination enhanced the cGAS-STING axis and downregulated Fas ligand (FasL) on M2 macrophages, thereby improving T-cell survival and mediating abscopal tumor regression (62).

Resistance to PD-L1 blockade in NSCLC has also been addressed through macrophage modulation. While hypofractionated radiation alone produced mixed macrophage phenotypes, the addition of a Src homology region 2 domain-containing phosphatase-2 (SHP-2) inhibitor to radiotherapy and anti-PD-L1 treatment significantly shifted polarization toward an M1 state (CD^38+^, CD^68+^). Notably, SHP-2 expression was elevated in M1 TAMs following radiation, making them susceptible to pharmacological reprogramming that subsequently improved local control and reduced lung metastases (63).

In TNBC, the combination of radiotherapy, PD-L1 blockade, and CDK4/6 inhibition (Abemaciclib) demonstrated that cell-cycle modulators can also influence myeloid biology. While radiation alone resulted in modest macrophage changes, the triple therapy induced a robust increase in MCP-1+, CD^80+^, and iNOS^+^ M1 macrophages with a concurrent decrease in CD^206+^ M2 cells. This polarization aligned with maximal T-cell infiltration and tumor growth inhibition (64).

Advanced delivery systems have further exploited these mechanisms. A multifunctional *in situ* vaccine strategy, combining Fe3O4-based nanoparticle radiosensitizers, a Toll-like receptor 7 (TLR7) agonist, and Cytotoxic T-lymphocyte-associated protein 4 (CTLA-4) blockade, amplified radiation-induced ROS and reshaped the microenvironment. The regimen successfully shifted macrophage populations from M2 to M1, a change that correlated with potent local control and the regression of distant untreated tumors (65).

Beyond checkpoint blockade, radiation-induced macrophage plasticity serves as a conditioning regimen for adoptive cell therapies. In pancreatic ductal adenocarcinoma, a single dose of local radiation rapidly altered the myeloid landscape, increasing M1-associated signatures that peaked at 48 hours. When followed by CLDN18.2-targeted CAR-T therapy, this conditioning led to a sustained M1-dominant environment (high iNOS, low CD^206^) and enhanced CAR-T cell infiltration and effector function, significantly delaying tumor growth compared to CAR-T therapy alone (66).

Conversely, the choice of combinatorial agents can hinder favorable macrophage remodeling. In glioma models, radiation alone successfully reduced immunosuppressive M2 TAMs, which correlated with better outcomes. However, the addition of temozolomide created a suppressive immune profile that negated these benefits, and the subsequent addition of anti-PD-1 failed to restore cytotoxic immunity. This highlights that standard chemotherapeutics may interfere with the pro-inflammatory macrophage shifts induced by radiation (67).

In these models, ROS enhancement appears beneficial because it is coupled to immunogenic tumor damage and inflammatory macrophage reprogramming; this differs from settings in which persistent oxidative or lipid-peroxidation signaling sustains immunosuppressive macrophage states. Collectively, these studies underscore that radiotherapy functions as a potent modulator of macrophage polarization, capable of priming the TME for immunotherapy. Whether through direct synergy with checkpoint inhibitors, targeted modulation of resistance pathways, or preconditioning for CAR-T cells, the therapeutic efficacy of these combinations is frequently underpinned by a shift from M2- to M1-like macrophage phenotypes. This repolarization is not merely a marker of response but a functional prerequisite for recruiting T cells, sustaining adaptive immunity, and mediating systemic abscopal effects.

### 4.2 Targeting recruitment and survival

Because radiotherapy can increase the recruitment and persistence of immunosuppressive TAMs, several studies have evaluated whether interrupting these pathways can improve treatment response. Overall, the evidence suggests that blocking macrophage recruitment, survival, or function can limit radiotherapy-induced immunosuppression, promote a more inflammatory macrophage phenotype, and enhance tumor control.

Chemokine-driven recruitment is one important mechanism. In a murine Lewis lung carcinoma model, hypofractionated radiotherapy induced early remodeling of the tumor microenvironment by expanding a subset of M2-like macrophages characterized by high C-C motif chemokine ligand 8 (CCL8) expression. Radiotherapy also increased circulating CCL2, CCL7, and CCL8, thereby reinforcing macrophage recruitment and establishing a feed-forward loop of TAM accumulation. Pharmacologic inhibition of CCL signaling with Bindarit reduced infiltration of M2-like macrophages and improved tumor control, indicating that chemokine blockade can counteract radiotherapy-induced macrophage-mediated immunosuppression (68). A related mechanism has been described in hepatoma, where radiotherapy promoted M2 polarization through the CCL5-CCR5 axis. In this setting, the CCR5 antagonist maraviroc reversed the M2-biased phenotype, increased M1-like polarization, and enhanced radiosensitivity, further supporting chemokine-targeted strategies as a means of improving radiotherapy response (69).

The colony-stimulating factor 1 receptor (CSF-1R) pathway represents another major target for limiting macrophage support of tumor regrowth after irradiation. In glioblastoma models, radiotherapy increased CSF-1R ligands and promoted the recruitment and maintenance of M2-polarized TAMs. CSF-1R blockade with PLX3397 reversed this shift, reduced macrophage infiltration, suppressed angiogenesis, and enhanced the therapeutic effect of radiotherapy, resulting in greater tumor regression and improved survival (70). Similar results were observed with BLZ-945, which also reduced M2 polarization and strengthened radiotherapy-induced antitumor immunity in glioblastoma models (71). Together, these studies suggest that CSF-1R inhibition can disrupt a key survival and maintenance pathway for protumor macrophages in irradiated tumors.

Other approaches aim less at recruitment itself and more at macrophage function in the post-radiotherapy setting. In a syngeneic MC38 colorectal cancer model, radiotherapy combined with dual checkpoint blockade targeting CD^47^/SIRPα and PD-1/PD-L1 increased CD^11c+^ M1-like macrophages, reduced CD^206+^ M2-like macrophages, enhanced macrophage phagocytosis, and strengthened T-cell activation. This combination produced greater tumor inhibition than radiotherapy alone, suggesting that macrophage-directed checkpoint blockade can amplify both innate and adaptive antitumor immunity after irradiation (72). Likewise, in lung cancer, radiotherapy-induced MerTK-dependent efferocytosis promoted M2-like polarization, impaired antigen presentation, and limited abscopal responses. Inhibition of MerTK with antisense oligonucleotides increased the M1/M2 ratio, restored antigen presentation, and improved sensitivity to checkpoint blockade, indicating that efferocytosis pathways can also be exploited to reprogram macrophages after radiotherapy (73).

These studies show that macrophage-directed strategies can enhance radiotherapy through several complementary mechanisms. Blocking chemokine axes such as CCL2/7/8 and CCL5-CCR5 can reduce recruitment of immunosuppressive macrophages, CSF-1R inhibition can limit their survival and maintenance, and functional targeting of CD47/SIRPα or MerTK can restore phagocytosis and antigen presentation. Across models, these interventions consistently shift macrophages away from an M2-like state and toward a more inflammatory phenotype, thereby improving radiotherapy efficacy and supporting combination immunotherapy approaches.

### 4.3 Metabolic and small molecule reprogramming

A growing body of work suggests that radiotherapy can be potentiated by small molecules that reprogram macrophages away from an immunosuppressive phenotype and toward a more inflammatory, tumor-supportive state. Although these agents act through different pathways, most converge on a similar outcome: reduced M2-like polarization, enhanced antigen presentation or phagocytic activity, stronger CD^8+^ T-cell responses, and improved tumor control.

Several studies have focused on pathways that connect tumor cell stress responses to macrophage activation. Ma et al. (74) showed that combining radiotherapy with the B-cell lymphoma 2 (BCL-2) inhibitor Sonrotoclax promoted macrophage reprogramming toward an M1-like phenotype through ferroptosis-associated damage signals and activation of the cGAS-STING pathway. This was accompanied by stronger CD^8+^ T-cell responses and improved tumor control, suggesting that BCL-2 inhibition can amplify both innate and adaptive immunity after irradiation. In head and neck squamous cell carcinoma, Moreira et al. (75) identified STAT3 as a key driver of radiation-associated M2 polarization. Inhibiting this pathway with a CpG-STAT3 antisense oligonucleotide shifted macrophages toward an M1-like phenotype, increased CD8+ T-cell activation, and improved tumor control and survival, supporting myeloid STAT3 as another candidate target for overcoming radiation-induced immunosuppression.

Other small-molecule strategies target signaling pathways that sustain protumor macrophage function after radiotherapy. HDAC6 inhibition has been reported to counteract radiation-induced M2 polarization and preserve a more favorable M1/M2 balance, thereby enhancing antitumor immunity when combined with radiotherapy (76). In pancreatic cancer, inhibition of RAGE signaling similarly reduced M2-like macrophages and increased CD8+ T-cell infiltration, highlighting the contribution of macrophage reprogramming to reversal of radioresistance in this setting (77). Rapamycin provides a somewhat different example, as its main effect appears to be functional rather than purely phenotypic: induction of autophagy in M2 macrophages reduced their protumor secretory program and increased tumor apoptosis after radiotherapy, indicating that radiosensitization may be achieved not only by changing macrophage identity but also by altering macrophage behavior (78).

Vascular and metabolic normalization may also contribute to macrophage-directed radiosensitization. Yuan et al. (79) found that although radiotherapy alone increased immunosuppressive myeloid populations, addition of the antiangiogenic agent Famitinib normalized the vasculature, reduced hypoxia, promoted M1-like polarization, and enhanced T-cell infiltration and tumor control. These findings suggest that part of the benefit of antiangiogenic therapy in combination with radiotherapy may derive from indirect remodeling of macrophage polarization through changes in the tumor microenvironment.

Additional compounds further support this general principle. Magnolol enhanced M1-like polarization by inhibiting EGFR and NF-κB signaling, whereas valproic acid-like agents reversed radiation-induced M2 polarization, strengthened T-cell responses, and improved tumor control (80, 81). Radiation-induced phosphatidylserine exposure represents another actionable pathway. Budhu et al. showed that blocking phosphatidylserine signaling with mch1N11 during radiotherapy redirected macrophages toward an M1-like phenotype, improved T-cell priming, and enhanced treatment efficacy, again underscoring the importance of macrophage reprogramming as a determinant of radiation response (81).

These studies underscore the crucial role of macrophage polarization in the effectiveness of radiotherapy. The combination of radiation therapy with agents that influence macrophage phenotype, whether through metabolic pathways, genetic modulation, or targeted inhibitors, presents a promising strategy for overcoming the immunosuppressive and radioresistant properties of the TME. These findings pave the way for future therapies aimed at reprogramming macrophages to enhance antitumor immunity and improve clinical responses to radiotherapy.

### 4.4 Immunostimulants: vaccines and STING agonists

Radiotherapy, combined with immunostimulants such as STING agonists, vaccines, and TLR agonists, can reshape macrophage polarization within the TME, significantly impacting the efficacy of cancer treatments. Several studies demonstrate how these combinations alter macrophage populations, from pro-tumorigenic M2 macrophages to pro-inflammatory M1 macrophages, thereby enhancing the immune response to radiotherapy.

One of the clearest examples is the combination of radiotherapy with STING agonism. In preclinical models, this strategy increased CD^86^-positive macrophages and reduced CD^206^-positive macrophages, indicating a shift toward an M1-like phenotype. This macrophage reprogramming was accompanied by greater infiltration of CD^8+^ T cells, dendritic cells, and natural killer cells, as well as improved local and distant tumor control, including a pronounced abscopal effect. These findings support the idea that activation of innate immune sensing pathways can amplify the immunogenic effects of radiotherapy in part through macrophage remodeling (82).

Vaccine-based approaches have shown similar effects. In one study, a fibroblast activation protein-alpha (FAPα)-targeted cancer vaccine combined with stereotactic ablative radiotherapy reduced stromal support, limited fibrosis, and shifted tumor-associated macrophages from an M2-like to an M1-like phenotype. This was associated with improved tumor control, reduced metastasis, and prolonged survival, suggesting that stromal targeting can complement radiotherapy not only by affecting fibroblasts but also by indirectly reprogramming macrophages (83). Likewise, brachytherapy has been explored as a form of *in situ* vaccination, with evidence that dose influences the degree of macrophage repolarization. In that model, a 10-Gy dose produced the most marked reduction in M2-like macrophages, the greatest increase in cytotoxic T-cell infiltration, and the strongest tumor growth inhibition, indicating that immunostimulatory effects of radiotherapy may depend partly on achieving a dose threshold for macrophage remodeling (84).

Other immunostimulatory strategies also appear to enhance radiotherapy by activating macrophages more directly. In prostate cancer models, the CD^40^ agonist HERA-CD^40^L repolarized macrophages toward an M1-like phenotype and, when combined with radiotherapy, increased T-cell infiltration and improved antitumor activity. These results suggest that CD^40^ signaling can help overcome radiation-associated immune suppression by strengthening both myeloid and lymphoid responses (85). Microbial platforms have produced comparable findings. In an intracranial sarcoma model, engineered Salmonella typhimurium secreting flagellin B increased M1-like macrophage polarization when combined with radiotherapy, leading to greater tumor regression and longer survival (86). In melanoma, attenuated Salmonella delivering IL-21 reduced M2 macrophage infiltration, enhanced cytotoxic T-cell activity, and improved tumor regression and apoptosis after radiotherapy, again highlighting the importance of macrophage repolarization in the therapeutic response (87).

Taken together, these studies indicate that immunostimulatory agents can enhance radiotherapy through a shared immunologic mechanism: reprogramming macrophages toward a more inflammatory and tumoricidal phenotype while strengthening downstream adaptive immunity. Although the agents differ in their proximal targets, STING agonists, vaccines, CD40 agonists, and microbial delivery platforms all appear capable of shifting the balance away from M2-like macrophage dominance and toward a microenvironment more permissive to effective tumor control. These findings support immunostimulatory macrophage reprogramming as a promising strategy for improving radiotherapy outcomes.

## 5 Bioengineering the niche: nanotechnology and biomaterials

### 5.1 Multifunctional nanoparticles for radio-immunotherapy

In recent studies, the combination of radiotherapy with nanoparticles to modulate macrophage polarization has emerged as a promising strategy for enhancing radio-immunotherapy. **Table 2** summarizes current studies on this matter.

**Table 2:** Use of functionalized nanoparticles in combination with radiotherapy to modulate macrophage polarization and enhance tumor immunotherapy.

Nanoparticles	Cancer model	Macrophage polarization	Tumor response	Mechanisms	Ref.
Biofunctionalized Liposome-like Nanovesicles (BLNs) + anti-PD-1 + RT	Lung cancer (LLC), Melanoma (B16-F10)	M1↑, M2↓ (via MAPK pathway, increased CD86, iNOS, Stat1)	Enhanced T-cell infiltration, improved survival, synergy with PD-1 blockade	BLNs induce M1 polarization via dsDNA cargo, promote phagocytosis, immune cell activation	(88)
NBTXR3 Hafnium Oxide Nanoparticles + Proton Radiotherapy (PRT) + anti-PD-1	Lung cancer (344SQR)	M1↑ (Nos2, glycolysis genes), M2↓	Improved tumor control, abscopal effect, 40% cure rate with NBTXR3 + PRT	PRT + NBTXR3 enhances M1 polarization, glycolytic shifts, cytokine release, immune remodeling	(89)
Mannose-levamisole Co-loaded PEGylated Liposomes (M/LM-Lipo) + RT	Pancreatic cancer (PANC02)	M2↓ in both local and distal tumors	Complete tumor eradication, strong abscopal effect, enhanced T-cell infiltration	M/LM-Lipo reprograms M2 macrophages, alters metabolism, enhances immune responses	(90)
Polymer-Lipid-Manganese Dioxide Nanoparticles (PLMDs) + RT	Prostate cancer (PC3, TRAMP-C2)	M1↑, M2↓ (hypoxia reversal, increased iNOS, CD86)	Increased apoptosis, delayed tumor growth, improved survival	PLMDs reduce hypoxia, activate M1 polarization, reduce immunosuppressive M2 signals	(91)
RGD-modified Liposomes Encapsulating ROCK Inhibitor Y-27632 (RGD@LP-Y) + RT	Hepatocellular carcinoma (Hepa1-6)	M1↑ (CD86), strong immune activation	Strongest tumor inhibition, survival improvement	RGD@LP-Y modulates matrix stiffness, activates PI3K/AKT/NF-κB, enhances ROS and ICD	(92)
Nanoscale Metal-Organic Framework (nMOF) Hf-DBP + TLR7 Agonist Imiquimod (IMD) + Anti-CD47 Antibody (αCD47) + RT	Colorectal cancer (CT26)	M1↑, M2↓ (repolarization with TLR7 agonist and αCD47)	Near-complete tumor eradication, strong abscopal effect, PD-L1 synergy	ROS generation, immunogenic cell death, enhanced macrophage phagocytosis, antigen presentation	(93)
Bismuth-based Nanoscale Metal-Organic Framework (Bi-DBP) + RT + Anti-PD-L1	Prostate/Pancreatic cancer (TRAMP-C2/Panc02)	M1↑, M2↓ (via ROS, lower TGF-β, ECM remodeling)	Enhanced RT response, abscopal regression, improved T-cell infiltration	Bi-DBP amplifies ROS, repolarizes M2→M1, reduces TGF-β, enhances T-cell entry	(94)
Cerium Oxide Nanoparticles Encapsulated in Bacterial Outer Membrane Vesicles (CeO_2_@OMV) + RT	Triple-negative breast cancer (4T1)	M1↑, M2↓ (iNOS↑, TNF-α↑)	Strongest inhibition of primary and distant tumors, prevention of lung metastasis	CeO_2_@OMVs increase ROS, induce immunogenic cell death, activate M1 macrophages for systemic antitumor effects	(95)
Catalase–Gold Nanoaggregates (Au@CAT) + RT	Colorectal cancer (CT26)	M1↑, M2↓ (shift in M1/M2 ratio, CD86↑, iNOS↑)	Maximal tumor suppression, prolonged survival, reduced lung metastasis	Au@CAT relieves hypoxia, enhances ROS, shifts M2 macrophages to M1, reduces immunosuppressive cells	(96)

RT, radiotherapy.

Biofunctionalized liposome-like nanovesicles (BLNs) derived from irradiated cancer cells are shown to efficiently repolarize TAMs from an M2-like to an M1-like phenotype. This repolarization is associated with increased expression of pro-inflammatory markers such as CD^86^, Nos2, and Il12a, which enhances macrophage-mediated antitumor immunity. Additionally, BLNs enhance the effectiveness of PD-1 blockade therapy by facilitating robust CD^8+^ T-cell–dependent tumor rejection. This study highlights the potential of irradiated-cell-derived nanovesicles in reproducing radiotherapy’s immunomodulatory effects without delivering radiation directly to the tumor (88).

A study involving proton-based radiotherapy combined with the NBTXR3 nanoparticle radioenhancer reveals that proton therapy more efficiently reprograms TAMs toward an M1 phenotype compared to photon-based treatments. NBTXR3-augmented proton therapy not only enhances macrophage polarization but also improves tumor control, both locally and systemically, with the highest induction of M1 macrophages correlating with superior tumor regression. This approach significantly reduces tumor metastasis and shows promise in overcoming resistance in immune checkpoint–resistant lung cancer models (89).

The role of exosomes in macrophage polarization is further explored in a study involving lung cancer cells. After 10 Gy X-ray irradiation, tumor-derived exosomes increase macrophage proliferation and induce a cytokine response skewed toward M2 polarization, primarily through miR-4655-5p. This study underscores the importance of exosomal communication in the modulation of macrophage phenotypes and the potential challenges of radiation-induced immunosuppression, as M2 macrophages contribute to tumor progression and immune evasion (90).

A multifunctional nanocomposite developed by Peng et al. also demonstrates enhanced radiotherapy response by targeting macrophages in breast cancer. By promoting mitochondrial ROS production and activating the cGAS-STING pathway, this nanocomposite triggers macrophage reprogramming from an M2 to an M1 phenotype. This shift correlates with improved tumor control, reduced hypoxia, and heightened immune activation, including increased infiltration of CD^8+^ T cells. The combination of radiotherapy and nanocomposite treatment significantly enhances tumor suppression and induces immune memory (97).

Another study utilizes metabolic targeting to reprogram macrophage polarization in response to radiation-induced immunosuppression. Combining mannose and levamisole in PEGylated liposomes effectively reverses the M2 polarization induced by irradiated tumor-derived extracellular vesicles. This dual inhibition of glycolysis and mitochondrial maintenance enhances antigen presentation T-cell activation, leading to complete tumor ablation and an abscopal effect, demonstrating the importance of metabolic modulation in reversing radiation-induced immune suppression (98).

Research by Zetrini et al. examines how MnO_2_-based nanoparticles modulate the TME by alleviating hypoxia and reshaping macrophage polarization. These nanoparticles, when combined with radiotherapy, shift TAMs from an M2 to an M1 phenotype, which is associated with increased T-cell infiltration and improved tumor control. This approach highlights the importance of microenvironment normalization in enhancing the radiotherapeutic response and overcoming radioresistance (91).

ROCK inhibition delivered via RGD-modified liposomes also enhances radiotherapy efficacy by reprogramming the immunosuppressive tumor matrix. This study shows that softening the extracellular matrix and suppressing YAP/COL1A2 expression increase pro-inflammatory macrophage polarization, improving radiation-induced DNA damage and immunogenic cell death, thus enhancing tumor suppression (92).

Nanoscale metal-organic frameworks (MOFs) have also been explored for macrophage-targeted immunomodulation in colorectal cancer. These MOFs, when combined with radiotherapy, repolarize TAMs from an M2 to an M1 phenotype, facilitating enhanced antigen presentation and CD^8+^ T-cell infiltration. This approach leads to significant tumor regression and suggests a potential strategy for overcoming immune evasion in tumor models resistant to traditional therapies (93).

Similarly, the use of a Bi-based nanoscale MOF in prostate and pancreatic tumors enhances radiotherapy efficacy, leading to M2-to-M1 polarization of TAMs and improved antitumor immunity. When combined with PD-L1 blockade, this therapy induces robust immune responses, resulting in substantial tumor regression and abscopal effects (94).

A study on cerium oxide-loaded bacterial outer membrane vesicles (CeO_2_@OMVs) further confirms that radiation-induced immunogenic cell death can be harnessed to reprogram macrophages. In TNBC, CeO_2_@OMVs facilitate M1 polarization of macrophages and enhance systemic immune responses, contributing to improved tumor control and metastasis prevention (95).

Finally, catalase–gold nanoaggregates have been shown to enhance radiation-induced immunomodulation in colorectal cancer. By relieving hypoxia, these nanoparticles shift the macrophage population toward an M1 phenotype, thereby amplifying the antitumor immune response and improving therapeutic efficacy (96).

In summary, these studies demonstrate the substantial potential of combining radiotherapy with nanoparticles to modulate macrophage polarization and enhance the immune response against tumors. The reprogramming of TAMs from an immunosuppressive M2 phenotype to a pro-inflammatory M1 phenotype is consistently associated with improved tumor control, enhanced immune activation, and superior therapeutic outcomes. These findings underscore the growing importance of macrophage-targeted strategies in overcoming the limitations of traditional cancer therapies and improving the efficacy of radio-immunotherapy.

### 5.2 Innovative delivery mechanisms and radiosensitizers

Recent delivery platforms and radiosensitizers increasingly exploit macrophages not only as therapeutic targets but also as carriers, sensors, and amplifiers of radiotherapy-induced immune responses. Although these approaches differ in composition and mechanism, many converge on a common principle: improving radiotherapy efficacy by shifting macrophages away from an immunosuppressive state and toward a more inflammatory, tumor-controlling phenotype.

One strategy uses radiation-induced myeloid trafficking to improve drug delivery. In glioma, Kuang et al. showed that a single 5-Gy dose increased CCL2 expression, recruited monocytes into the tumor, and promoted a more M1-like macrophage milieu. This environment enabled D@MLL nanoparticles to attach to infiltrating monocytes and release doxorubicin within the MMP-2-rich tumor niche. The resulting immunogenic cell death further reinforced M1 polarization, promoted dendritic cell maturation, and enhanced CD^8+^ T-cell activation, leading to better tumor control than either treatment alone (99). A conceptually related approach targeted hypoxia rather than trafficking. In the BFO/BWO-PVP nanoplatform, light activation relieved tumor hypoxia, suppressed HIF-1α signaling, and favored an M2-like to M1-like phenotype shift. When combined with radiotherapy, this oxygenation-driven reprogramming was associated with higher ROS, greater antigen presentation, increased CD^8+^ T-cell infiltration, reduced metastasis, and improved survival, indicating that correction of hypoxia can create conditions that support macrophage-dependent radiosensitization (100).

Other platforms enhance radiotherapy by coupling immunogenic tumor cell death to local macrophage reprogramming. Luo et al. (101) described a phosphorylated peptide system that self-assembled into an intratumoral nanofiber matrix after radiotherapy-induced tumor damage, thereby trapping tumor antigens and functioning as an *in-situ* vaccine. Because the construct also contained flurbiprofen, it inhibited COX-2 signaling and reduced M2 polarization, as reflected by increased CD86 *in vitro* and increased iNOS with reduced Arg-1 *in vivo*. These changes were accompanied by greater T-cell infiltration and stronger tumor control than either component alone. A similar immunogenic mechanism was reported with the bismuth-containing UCNP-DOX radiosensitizer. In that study (102), radiotherapy alone favored an M2-like cytokine profile, whereas addition of UCNP-DOX induced immunogenic cell death and redirected macrophages toward an M1-like phenotype, with increased CD86/CD68 and higher secretion of IL-12p70, GM-CSF, IFNγ, and TNF-β. *In vivo*, this was associated with M1 enrichment, increased CD8+ T-cell infiltration, and near-complete tumor control. Likewise, hafnium-silica nanoparticles carrying SiPCCl2 enhanced X-ray-induced electron-dynamic therapy, triggering calreticulin, HMGB1, and ATP release, promoting dendritic cell activation and CD^8+^ priming, and increasing M1 while reducing M2 macrophages in both irradiated and distant tumors. These changes were accompanied by improved local control and abscopal activity, with proteomic evidence of inflammatory and interferon signaling consistent with M1 polarization (103). Mn porphyrin BMX-001 (MnBuOE) produced a related myeloid shift in 4T1 tumors, where the combination of MnBuOE and radiotherapy increased M1-associated transcripts such as Il1a, Il1b, Cxcl3, Mmp12, Ccl3, and Ccl4 while reducing Arg1, CD206, and IL10, resulting in a more favorable CD86/CD206 balance and decreased myeloid-derived suppressor cells (104).

Not all delivery systems, however, produce the intended macrophage response on their own. In melanoma, low-dose ^177^Lu-DOTA-CEMJ4 radionuclide therapy increased T-cell infiltration but did not meaningfully repolarize macrophages and also suppressed T-cell activation. Only when combined with an HPK1 inhibitor did this strategy restore T-cell function and increase M1 macrophage accumulation, indicating that some radionuclide-based approaches require additional immune modulation to achieve macrophage-dependent benefit (105). Ferumoxytol provided an even more cautionary example. In a 4T1 dual-tumor model, neither radiotherapy alone nor radiotherapy plus ferumoxytol promoted M1 polarization; instead, ferumoxytol increased M2 proportions and reduced MHC-II expression on macrophages and dendritic cells. Although the therapeutic effect was limited, ferumoxytol uptake still served as an imaging readout of macrophage behavior, suggesting that some platforms may be more valuable for monitoring myeloid responses than for directly improving them (106).

Taken together, these studies show that innovative delivery systems and radiosensitizers can substantially influence radiotherapy outcomes through macrophage biology. Monocyte-hitchhiking nanoparticles, hypoxia-correcting catalysts, *in situ* vaccine platforms, immunogenic radiosensitizers, and redox-active agents generally enhance radiotherapy by promoting M1-like polarization and strengthening downstream adaptive immunity (99–104). By contrast, ferumoxytol and radionuclide therapy alone illustrate that macrophage responses are not uniformly favorable and may require additional immune-directed intervention (105, 106). Overall, these findings reinforce the idea that successful radiotherapy-adjacent technologies depend not only on drug delivery or physical dose enhancement but also on how effectively they reshape the macrophage compartment.

## 6 Systemic implications: abscopal effects and normal tissue toxicity

### 6.1 Mediating the abscopal effect and systemic immunity

The available preclinical evidence suggests that macrophage polarization is a major determinant of whether radiotherapy generates only a local response or also induces systemic antitumor immunity. Across models, abscopal effects are more consistently observed when irradiation is accompanied by a shift toward M1-like macrophage states, whereas M2-dominant programs appear to limit distant tumor control and may even promote radioresistance.

One mechanism linking local irradiation to systemic immunity involves tumor-draining lymph nodes (TDLNs). Liu et al. showed that in models treated with a single 12-Gy fraction plus anti-PD-1, macrophages in both irradiated and distant tumors acquired a more inflammatory phenotype, with increased CD86 and MHC-II and reduced M2-associated markers. These changes coincided with greater CD^8+^ T-cell infiltration at both primary and abscopal sites. Removal of the TDLNs disrupted this coordination, drove macrophages toward an M2-like state, weakened chemokine and cytokine programs associated with M1 polarization, and diminished systemic tumor control, indicating that TDLNs help orchestrate macrophage reprogramming across tumor sites (107).

Pharmacologic modulation can similarly determine whether macrophage responses support distant tumor regression. In a DMBA-induced breast cancer model, radiotherapy alone increased macrophage recruitment to distant lesions but favored an M2-like phenotype characterized by higher CD^163^ and Arg-1, and no abscopal effect was observed. Adding the histone deacetylase inhibitor HPTA reversed this pattern: distant tumors showed increased CD^86^, iNOS, and pro-inflammatory cytokines, along with reduced immunosuppressive mediators, decreased proliferation, greater necrosis, and inhibition of distal tumor growth. These findings suggest that radiotherapy may require concurrent macrophage reprogramming to generate effective systemic immunity in some settings (108).

Tumor-derived vesicle signaling provides another route by which local irradiation can influence macrophages at distant sites. In a TP53-dependent model of single-fraction high-dose irradiation (20 Gy), senescent tumor cells released extracellular vesicles containing DNA: RNA hybrids and LINE-1 sequences that induced senescence in contralateral tumors and reprogrammed macrophages toward an M1-like phenotype. In both murine and human macrophage models, these vesicles increased IL-1β, IL-6, and STAT1 while reducing Arg1 and Egr2. Distant tumors developed senescence-associated changes only when the irradiated tumors retained functional p53, indicating that vesicle-mediated macrophage reprogramming can participate in long-range radiation signaling (109).

Not all macrophage responses are favorable for systemic control. The radiation-induced rescue effect (RIRE) illustrates how M2-like macrophages may blunt radiosensitivity rather than extend it. *In vitro*, M2 and tumor-associated macrophage-like populations reduced γH2AX and 53BP1 responses in irradiated cancer cells and amplified rescue signals from unirradiated bystander cells, whereas M1 macrophages showed little rescue activity and weakened signals generated by tumor cells or M2/TAM-like subsets. These findings imply that M2-rich environments may protect tumor cells from radiation damage, while M1-like conditions are more compatible with broad radiosensitization (110).

Dose and fractionation also influence whether macrophages support abscopal responses. Barsoumian et al. reported that low-dose radiation (1 Gy per fraction, delivered in two fractions) to lung tumors favored M1 enrichment and reduced TGF-β, whereas higher-dose regimens (12 Gy per fraction, delivered in three fractions) promoted a more suppressive stromal environment. Low-dose treatment alone did not produce a strong abscopal response, but when high-dose radiation was delivered to the primary tumor and low-dose radiation to a secondary lesion, the resulting “RadScopal” approach created a distal microenvironment more permissive to systemic immunity. In combination with PD-1 and CTLA-4 blockade, this regimen produced the strongest M1 polarization in distant tumors and involved coordinated NK- and CD4+ T-cell responses (111). A related dose-dependent pattern was observed in a syngeneic TNBC model, where intermediate low-dose radiotherapy (1.0-1.5 Gy) under PD-1 blockade reduced TGM2+ M2-like macrophages and expanded CD^68+^ macrophages with M1-like features in both irradiated and non-irradiated tumors. These doses produced the greatest tumor growth delay, whereas higher doses were associated with re-emergence of M2-like populations, suggesting that there may be a narrow dose window in which macrophage reprogramming best supports systemic antitumor activity (112).

Taken together, these studies indicate that the abscopal effect is not simply a consequence of local tumor irradiation, but rather depends on whether irradiation is able to establish a macrophage state that supports systemic immune activation. TDLN signaling, tumor-derived extracellular vesicles, pharmacologic reprogramming, and dose selection all influence this process. Across models, M1-like macrophage polarization is repeatedly associated with effective distant tumor control, whereas M2-dominant programs correlate with rescue signaling, immune suppression, and failure of systemic response. These findings position macrophage plasticity as a central link between local radiotherapy and whole-body antitumor immunity.

### 6.2 The microbiome-macrophage axis in radioprotection

Radiotherapy disrupts mucosal barriers and microbial communities, and these changes can strongly influence macrophage polarization during tissue injury and repair. Across oral, intestinal, and pelvic irradiation models, radiation-induced dysbiosis is often associated with inflammatory, M1-skewed macrophage responses that aggravate tissue damage, whereas interventions that restore microbial balance tend to favor M2-like programs linked to repair and radioprotection.

In the oral cavity, irradiation promoted microbial dysbiosis, increased LPS accumulation, and activated the TLR4/NF-κB pathway, driving macrophages toward an M1-like phenotype. This was reflected by increased iNOS-positive macrophages *in vivo* and higher CD^86^ expression in THP-1-derived macrophages *in vitro*, together with a broader pro-inflammatory cytokine response that worsened oral mucositis. Treatment with WC03 reduced these effects by stabilizing the microbial community, lowering LPS accumulation, and suppressing TLR4, MyD88, TRAF6, IRF5, NLRP3, and NF-κB p65 signaling. These changes were accompanied by reduced M1 polarization, while M2 levels remained relatively stable. Mechanistic experiments further suggested that Emodin was a major active component, limiting macrophage recruitment to irradiated epithelium and inhibiting TLR4 signaling in a dose-dependent manner (113).

A different but complementary pattern was observed in the gut, where abdominal irradiation impaired mucosal integrity and reduced M2-associated macrophage features, including CD206 and IL-10 expression. This loss of anti-inflammatory macrophages paralleled epithelial injury and increased inflammatory cytokines. Supplementation with Lactobacillus reuteri FN041 restored M2-like polarization, increased IL-10, and lowered IL-6 and TNF-a. At the same time, FN041 reshaped the gut microbiota toward taxa associated with short-chain fatty acid production and altered the metabolite profile in ways linked to M2-supportive programs, including changes in prostaglandins and phospholipid derivatives. These microbial and metabolic shifts coincided with improved mucosal repair after irradiation (114).

Microbiota-responsive drug delivery systems provide another way to exploit this axis therapeutically. After colonic irradiation, lamina propria macrophages accumulated in an M1-dominant pattern alongside dysbiosis, oxidative stress, and inflammatory cytokine release. CS/PEC-AMF nanoparticles, designed to release Amifostine selectively in the colorectum, reduced ROS, preserved epithelial structure, and promoted a shift from M1-like to M2-like macrophage polarization in both acute and chronic injury models. Transcriptomic analyses showed suppression of IL-17, TNF, NF-κB, and NOD-like receptor signaling together with increased DNA repair pathways. Notably, these protective effects did not compromise tumor control in colorectal cancer models (115).

A related radioprotective pattern was reported in pelvic irradiation models. Fractionated X-irradiation reduced total CD^68+^ and CD^163+^ macrophages in the bladder, and AA2G supplementation did not alter this response or interfere with tumor elimination. In the ileum, however, irradiation preserved CD^68+^ macrophages while reducing CD^163+^ M2-like cells, creating an imbalance associated with mucosal injury. AA2G shifted this balance toward an M2-dominant state, decreasing CD^68+^ cells, increasing CD^163+^ macrophages, and improving the CD^163+^/CD^68+^ ratio in parallel with preservation of villus structure. These findings suggest that AA2G can support tissue-protective macrophage responses in irradiated intestine without reducing antitumor efficacy (116).

Importantly, these observations also highlight that M2-like macrophage functions should not be viewed as uniformly detrimental in the context of radiotherapy. While M2-like TAM programs within tumors are frequently associated with immunosuppression, radioresistance, and tumor progression, macrophages with anti-inflammatory and tissue-repair functions may be essential for limiting radiation-induced normal tissue injury and restoring epithelial integrity at mucosal sites. This distinction has direct translational implications for the therapeutic window. Strategies designed to deplete macrophages or enforce a strongly M1-like state may improve tumor radiosensitivity in some settings, but they could also impair normal tissue recovery or exacerbate treatment-related toxicity if applied without spatial or temporal selectivity. Conversely, the studies discussed here suggest that it may be possible to preserve or restore repair-associated macrophage programs in normal tissues without compromising tumor control, although current evidence remains largely preclinical. A clinically useful macrophage-targeted radiotherapy strategy will therefore likely need to account for this duality rather than assuming that all M2-associated functions are undesirable.

Taken together, these studies show that the microbiome and macrophage polarization are tightly linked during radiation-induced normal tissue injury. Dysbiosis and microbial products such as LPS tend to amplify inflammatory M1-like responses and worsen tissue damage, whereas microbial, phytochemical, nanoparticle-based, or metabolic interventions that restore microbial balance more often support M2-like repair programs. This microbiome-macrophage axis therefore represents an important component of radioprotection and a potential target for reducing treatment-related toxicity without compromising tumor control.

## 7 Translational insights: biomarkers and prognostic stratification

### 7.1 Predictive biomarkers and gene signatures

Macrophage-centered biomarkers and gene signatures linked to radiotherapy response have been defined through a range of experimental and clinical approaches, each offering a distinct window into how myeloid states forecast therapeutic sensitivity.

In nasopharyngeal carcinoma, Guo et al. showed that exosomal PTEN acts as a macrophage-modulating determinant of RT + IT efficacy, with PTEN-enriched exosomes suppressing M2 polarization and reinforcing M1 activation. When PTEN was depleted from exosomes, this shift was lost and M2 macrophages accumulated alongside weakened CD^8+^ T-cell activity and poorer tumor control, positioning exosomal PTEN as a macrophage-linked prognostic factor for combined-modality radiotherapy (117).

In cervical cancer, two transcriptomic studies independently identified macrophage-related immune signatures associated with radioresistance. Wu et al. developed a 14-gene immune risk score, finding that high-risk tumors—more likely to resist radiotherapy—were enriched for M0 and M2 macrophage infiltration. Although mechanistic pathways were not tested, the association between M2-dominant immune landscapes and treatment failure was consistent across analyses (118).

A related gene-signature analysis again linked persistent cervical tumors to higher M0 and M2 macrophage infiltration, with enrichment of pathways such as PI3K–Akt and MAPK signaling in radioresistant cases. This study did not observe shifts toward M1 states, instead identifying macrophage-associated immunosuppression as a transcriptional correlate of poor outcome (119).

In esophageal adenocarcinoma, pretreatment biopsies revealed that complete responders to chemoradiation exhibited lower infiltration of both M1 and M2 macrophages, whereas incomplete responders displayed a more macrophage-rich microenvironment. Subgroups defined by inflammatory and stromal programs differed in their macrophage-associated signatures, though the study remained associative and did not identify discrete polarization-specific predictors (120).

A radiotherapy-containing regimen for nasal NKTCL provided another angle, as patients who failed to clear EBV DNA during therapy had reduced monocyte and M1 macrophage infiltration. While macrophages were not the primary endpoint, this pattern suggested that insufficient M1-oriented immunity accompanied poorer treatment response (121).

In melanoma brain metastases, macrophage polarization emerged through an interferon-linked signature. Type I IFN signaling induced M1-like macrophage activation in murine models, and human MBM samples previously exposed to radiotherapy showed that tumors with a myeloid IFN-response signature had higher M1/M2 ratios and improved survival after RT. This signature thus reflected a macrophage phenotype associated with favorable radiotherapy outcomes (122).

Glioblastoma offered yet another macrophage-associated metric through the radiosensitivity index (RSI). Radioresistant tumors, across both bulk and single-cell datasets, consistently exhibited enrichment of M2 macrophages and lower M1/M2 ratios. Additional transcriptional programs in recurrent tumors, including TREM1 and CCR5 signaling, marked altered macrophage states that tracked with therapeutic response. Patients with low RSI and low M2 infiltration experienced the most favorable outcomes, linking macrophage polarization profiles to intrinsic radiosensitivity (123).

Finally, cervical cancer samples exposed to high-dose radiation (8 Gy single fraction) demonstrated a CAF-driven increase in M2 macrophage polarization through a CCL2–CCR2–PPARγ axis. High intratumoral M2 content correlated with worse progression-free survival following radiotherapy, and irradiated CAFs promoted a transcriptional M2 program encompassing CD206, Arg1, MRC1, and CCL22. This stromal-myeloid signature thus marked a treatment-relevant macrophage phenotype associated with radioresistance (124).

Taken together, these studies describe a landscape in which macrophage-associated biomarkers—ranging from exosomal PTEN to interferon-induced signatures, immune-derived gene scores, and stromal-regulated M2 programs—serve as indicators of radiotherapy response. Each dataset highlights a specific macrophage-linked signature rather than a universal pattern, but across cancers and methodologies, macrophage polarization consistently appears as a measurable and prognostically relevant component of radiotherapy sensitivity.

### 7.2 Clinical monitoring and imaging

Radiotherapy-driven macrophage dynamics have become increasingly accessible to clinical and preclinical monitoring, and the studies included here trace a spectrum of imaging and biomarker readouts that capture how macrophage states evolve after treatment. In the 4T1 breast tumor model, radiation produced a dose-dependent transition from CD^206+^ M2 macrophages toward iNOS^+^ M1 phenotypes, and this polarization shift was accompanied by a measurable rise in nitric oxide within the TME. The NO-responsive USPIO nanoprobe translated this biochemical change into a detectable MRI signal, allowing direct visualization of macrophage repolarization following X-ray exposure. Higher M1/M2 ratios and stronger nanoprobe activation appeared in tumors receiving 3 Gy, where reduced tumor growth aligned closely with increased NO-linked MRI contrast. This work demonstrates that monitoring the biochemical products of macrophage activation—rather than cell abundance alone—can provide a noninvasive window into how effectively radiotherapy reshapes the myeloid compartment (125).

A different imaging perspective emerges from TSPO-directed PET in head and neck squamous cell carcinoma. Here, radiotherapy elevated [18F]F-DPA uptake in xenografts and cultured tumor cells, but immune profiling showed concurrent reductions in both M1 and M2 macrophage subsets. The enhanced PET signal persisted despite this loss of differentiated macrophages, and blocking and silencing studies confirmed that tracer uptake depended on TSPO expression within tumor cells themselves. These findings indicate that TSPO imaging after irradiation tracks tumor-intrinsic metabolic responses rather than macrophage polarization states, offering a complementary view of how radiation alters the TME without directly engaging macrophage activity (126).

Macrophage-related biomarkers can also be measured systemically, as illustrated in an observational study of patients receiving pelvic radiotherapy for localized prostate cancer. Peripheral immune monitoring revealed a temporal shift: CD^86+^ M1 macrophages declined by week 4, while CD^163+^, CD^206+^, and CD^204+^ M2 populations rose by week 12. This polarization pattern coincided with increased serum and urinary concentrations of M-CSF, HGF, and MIP-1α—cytokines associated with macrophage recruitment and tissue remodeling. Elevated baseline M-CSF correlated negatively with patient-reported quality of life, suggesting that early macrophage-supportive signaling parallels the development of acute radiation cystitis. Clinical monitoring of circulating macrophage phenotypes and cytokines therefore captures treatment-related normal tissue responses that imaging alone may not reveal (127).

In resected NSCLC specimens following neoadjuvant chemoradiotherapy, macrophage monitoring shifted from circulation to tissue-level pathology. CD^204+^ M2-like macrophage density correlated positively with viable tumor cell percentages, indicating that residual tumor burden aligns with preserved M2 infiltration. Radiotherapy reduced CD^4+^, CD^8+^, and CD^20+^ lymphocytes but did not diminish CD^204+^ macrophages, and high CD204^+^ density was linked with poorer survival outcomes specifically in the chemoradiotherapy cohort. These observations position CD^204+^ tumor-associated macrophages as clinically relevant correlates of tumor persistence after irradiation, and they extend macrophage monitoring into prognostic territory (128).

Across these studies, imaging and clinical monitoring trace distinct but interconnected aspects of macrophage behavior after radiotherapy. MRI nanoprobe detection of nitric oxide captures functional repolarization; TSPO PET reveals tumor-intrinsic metabolic shifts independent of macrophage changes; peripheral cytokines and macrophage markers reflect systemic tissue responses; and tumor-resident CD^204+^ macrophages align with pathological and clinical outcomes. Together, these approaches illustrate how radiotherapy-associated macrophage activity can be visualized or quantified across biological scales, forming a composite framework for clinical assessment in both tumors and normal tissues.

### 7.3 Treatment response in clinical and preclinical cohorts

Across clinical and experimental settings, radiotherapy and chemoradiotherapy consistently altered macrophage composition in ways that were tightly interwoven with patterns of treatment response, although the direction and magnitude of these changes varied across disease contexts. In resected pancreatic ductal adenocarcinoma, gemcitabine-based neoadjuvant chemoradiotherapy produced a sex-specific pattern of macrophage remodeling. Tumors from women demonstrated reduced expression of the M2-associated factors CCL2 and IL34 and showed lower infiltration of CD^163+^ and MRC1^+^ macrophages throughout tumor compartments. These differences coincided with improved survival in women receiving the same treatment and with a lower M2/total macrophage ratio accompanied by higher BAD expression in carcinoma regions, suggesting a stronger apoptotic response within the treated tumor bed (129).

In soft-tissue sarcoma, neoadjuvant radiotherapy with or without chemotherapy generated a distinct expansion of macrophage-associated populations. CD^68+^/CD^163+^ and CD^206+^ M2 macrophages increased after treatment, and transcriptomic profiling confirmed upregulation of canonical M2 markers. Yet these increases were not linked to treatment efficacy. Instead, outcomes were more closely associated with monocyte expansion, and post-RT tumors showed heightened expression of antigen-presentation and phagosome-associated genes. The sarcoma data therefore demonstrate that radiotherapy can augment both suppressive and antigen-processing elements of the myeloid compartment without establishing a direct relationship between M2 levels and pathological response (130).

A similar M1 to M2 bias appeared in rectal cancer within the ADORE study, where paired biopsies and post-CRT specimens revealed that chemoradiotherapy increased CD^68+^CD^206+^ macrophages while reducing the proportion of CD^68+^CD^206^- cells. This transition occurred alongside higher T-cell and dendritic-cell infiltration. Neither baseline macrophage profile nor post-treatment M1/M2 proportions predicted tumor regression or disease-free survival, but the shift toward M2-associated phenotypes aligned with broader observations that radiotherapy can favor suppressive myeloid programs even as it recruits effector lymphocytes (131).

Another rectal cancer cohort employing transcriptomic deconvolution echoed these findings. Neoadjuvant chemoradiation increased intratumoral M2 macrophage fractions together with CD^8+^ T-cell infiltration. The study also identified increased tumor mutational burden and enrichment of immune-activation signatures after treatment, creating a setting in which heightened immunogenicity coexisted with an expanded M2 myeloid presence. While the functional impact of this shift on radiotherapy efficacy was not directly assessed, the reproducible rise in M2 macrophages across paired samples underscored the stability of this response pattern in rectal cancer (132).

In contrast, radiotherapy in an orthotopic NS/CT-2A glioma model produced reductions rather than increases in M2-polarized macrophages. Here, RT diminished total tumor-associated macrophages, M2 TAMs, and monocytic MDSCs while enhancing CD^8+^ T-cell infiltration. Better-responding animals consistently exhibited lower M2 infiltration together with higher cytotoxic T-cell presence, directly linking the macrophage profile to treatment success. When RT was paired with temozolomide, overall survival improved, but the myeloid landscape became more suppressive and CD^8+^ T-cell levels declined, dampening the beneficial macrophage shifts associated with RT alone (67).

A second brain-focused model, examining Lewis lung carcinoma brain metastases, found that radiotherapy alone increased M2-dominant features—including higher Arg-1, CD^206^, and CXCR4 expression—and expanded both microglial and bone-marrow–derived macrophage compartments. The anti-angiogenic agent Endostar, however, reversed these RT-induced M2 tendencies, restoring M1 markers and suppressing CXCR4 upregulation. Endostar’s vascular-normalizing activity reduced hypoxia and enhanced the overall antitumor effect of RT, showing how adjunct therapy can counteract macrophage polarization patterns that emerge following irradiation (133).

Finally, work in the GL261-luc2 glioblastoma system demonstrated that macrophage polarization can be shaped indirectly by soluble mediators released from irradiated tumor cells. Direct tumor–macrophage contact did not modify activation markers, but conditioned medium from RT- or chemoradiation-treated cells upregulated MHC-II, CD^80^, CD^86^, PD-L1, and ICOS-L in M1-like macrophages and reduced CD^206^ expression in M2-like cells. These findings point to a treatment-dependent secretome capable of selectively activating M1-associated pathways while partially modulating M2 identity (134).

Taken together, these clinical and preclinical cohorts illustrate that radiotherapy’s influence on macrophage polarization is highly context-dependent. Some settings show increased M2 infiltration after treatment, others display M2 suppression, and in several cancers, macrophage changes coexist with shifts in T-cell engagement or tumor immunogenicity. Across these diverse models, treatment response correlates most consistently with the balance of macrophage states rather than any single direction of change, reflecting the complexity of macrophage–radiotherapy interactions and underscoring the need for strategies tailored to specific tumor ecosystems. These cohort data support the clinical relevance of macrophage remodeling after radiotherapy, but they also underscore a central translational challenge: prognostic association alone does not establish which macrophage-directed interventions can be delivered safely, selectively, and effectively in human tumors.

### 7.4 Clinical translational barriers and future directions

Despite the growing number of macrophage-associated biomarkers and preclinical radiosensitization strategies, translating TAM-directed radiotherapy concepts into clinical practice remains challenging. First, pharmacokinetic and delivery barriers are substantial. Many candidate agents must reach macrophages embedded within hypoxic, poorly perfused, and stromally dense tumor regions, where drug penetration is heterogeneous and may differ markedly from systemic exposure. Second, target specificity remains limited. Pathways frequently used to deplete, recruit, or reprogram TAMs, including CSF-1/CSF-1R and chemokine axes, are not exclusive to tumor-promoting macrophages and may also affect circulating monocytes or macrophage populations involved in host defense and normal tissue repair. This raises a third challenge: systemic toxicity and therapeutic-window narrowing. In the context of radiotherapy, broadly suppressing macrophage survival or enforcing inflammatory polarization could improve tumor control in some settings but also worsen mucosal injury, fibrosis, or other treatment-related toxicities if repair-associated macrophage functions are disrupted.

A further barrier is the gap between murine models and human tumors. Many preclinical studies rely on transplantable mouse models with relatively uniform genetics, simplified immune environments, and limited representation of the spatial and phenotypic diversity seen in human TAM populations. In addition, marker-defined M1/M2 states in mice do not always map cleanly onto human macrophage programs, complicating biomarker transfer and drug selection. For this reason, the most clinically useful path forward will likely involve biomarker-guided and context-specific strategies rather than universal TAM depletion or a simple “M2-to-M1 switch.” Future studies should prioritize paired pre- and post-radiotherapy human samples, spatial and single-cell profiling, and early-phase trials that integrate pharmacodynamic macrophage readouts with normal-tissue toxicity monitoring. Such approaches may help identify when TAM targeting should be delivered concurrently with radiotherapy, when it should be sequenced after irradiation, and which patient subsets are most likely to benefit.

## 8 Contextual determinants of radiation-induced macrophage polarization

A more systematic interpretation of the literature shows that radiation-induced macrophage polarization is shaped by several interacting contextual factors rather than by radiation exposure alone. One of the most important determinants is tumor type. Across the studies, glioma, pancreatic cancer, rectal cancer, breast cancer, head and neck squamous cell carcinoma, and hepatocellular carcinoma do not show identical macrophage responses after irradiation. In many immunosuppressive tumor microenvironments, radiation is followed by enrichment of M2-like macrophage features associated with tissue repair, immune suppression, and therapeutic resistance. However, in selected experimental settings, especially when specific modalities or combination approaches are used, radiotherapy can also promote M1-like polarization and support antitumor immune activation. These differences indicate that the biological identity of the tumor strongly influences whether radiotherapy drives inflammatory reprogramming or reinforces suppressive macrophage functions.

Tumor stage and progression status also appear to be critical. Early-stage lesions may retain a microenvironment that is more permissive to inflammatory macrophage activity, whereas advanced, hypoxic, fibrotic, or recurrent tumors are more likely to favor M2-like polarization. As tumors progress, increasing hypoxia, stromal remodeling, metabolic competition, and chronic inflammatory signaling can shift macrophages toward programs that support angiogenesis, matrix remodeling, and immune escape. This may explain why similar radiation strategies can produce different macrophage phenotypes depending on whether they are applied in relatively early disease or in more advanced and treatment-resistant settings.

Another major source of heterogeneity is the radiation regimen itself, including dose, fractionation schedule, and delivery pattern. Conventional fractionation and more prolonged exposure schedules are often associated with tissue-repair signaling, persistent inflammatory stress, and conditions that support M2-like macrophage accumulation. By contrast, hypofractionated or ablative regimens may in some settings induce stronger immunogenic tumor cell death and favor a more M1-like macrophage phenotype. Thus, the effect of radiotherapy on macrophage polarization depends not only on the total dose delivered, but also on how that dose is fractionated over time.

The physical modality of radiation may further influence macrophage behavior. Evidence discussed in this review suggests that proton therapy, carbon-ion radiotherapy, and ultra-high dose-rate approaches such as FLASH radiotherapy may induce macrophage responses that differ from those seen with conventional photon irradiation. In several preclinical models, these approaches appear more likely to preserve or promote inflammatory macrophage features while reducing some of the suppressive remodeling observed after standard radiation exposure. Although these findings still require broader validation, they support the view that the immunologic impact of radiotherapy is partly modality dependent.

In addition, macrophage polarization after irradiation is influenced by macrophage origin and the tissue in which the tumor develops. Tumor-associated macrophages are not a single uniform population; they may derive from tissue-resident macrophages or from circulating monocytes recruited into the tumor. These populations differ in developmental origin, transcriptional programs, responsiveness to cytokines, and persistence after treatment. Such differences are particularly relevant in organs with specialized resident macrophage populations, such as microglia in the brain or Kupffer-cell-related populations in the liver. As a result, identical radiation regimens may generate distinct macrophage outcomes in different organs because the baseline myeloid compartment is fundamentally different.

Closely related to macrophage origin is the influence of the local tissue niche. Organ-specific stromal architecture, vascular features, extracellular matrix composition, metabolic constraints, and the degree of hypoxia all shape macrophage function after radiation. In some tissues, radiation-induced inflammation may resolve toward antitumor immune activation, while in others it may preferentially evolve into a wound-healing and pro-fibrotic program. This helps explain why the same broad radiation concept can produce divergent effects in tumors arising in the brain, pancreas, liver, rectum, breast, or lung.

Beyond tumor- and tissue-related variables, patient-related and systemic factors may also modify macrophage polarization. Baseline inflammatory status, microbiome-related immune tone, coexisting medical conditions, treatment-related normal tissue injury, and concurrent systemic therapies can all influence how macrophages respond to irradiation. Even when direct evidence remains limited in some settings, these host-related variables are clinically important because they may alter both local tumor immunity and broader systemic immune responses during treatment. Their influence is especially relevant when interpreting translational studies and when comparing findings across patient populations with different backgrounds and comorbidities.

Finally, the combined treatment context must also be considered. Radiotherapy is increasingly delivered together with immune checkpoint inhibitors, CAR-T strategies, anti-angiogenic agents, and macrophage-targeting therapies. In these settings, macrophage polarization reflects the combined pressure of radiation-induced damage signals and pharmacologic immune modulation. In several studies, reprogramming toward a more inflammatory and antitumor macrophage phenotype appears more likely when radiotherapy is paired with treatments that block suppressive pathways or enhance immune activation. This supports the idea that macrophage-directed radiosensitization should be developed in a context-specific manner rather than assuming a uniform macrophage response to radiation alone.

Taken together, the literature indicates that radiation-induced macrophage polarization should be understood as the product of interacting tumor-related, treatment-related, tissue-specific, and host-specific determinants.

## 9 Toward a working predictive framework for radiation-induced macrophage polarization

Here, we propose a working, hypothesis-generating framework for predicting the direction of macrophage polarization after radiotherapy. We emphasize that this is not a validated clinical algorithm, but rather a synthesis-based model derived from recurring patterns across the preclinical and translational literature reviewed herein. In this framework, radiation parameters act as upstream determinants, while tumor-specific and microenvironment-specific features determine how those signals are interpreted. Thus, post-radiotherapy macrophage polarization should be understood not as a fixed consequence of irradiation, but as the net result of interacting treatment-related, tumor-related, and ecological cues.

At the first level, radiation-related variables, including dose, fractionation, dose rate, and radiation quality, influence the balance between immunogenic cell death and tissue-repair signaling. At the second level, tumor context, including tumor type, disease stage, baseline myeloid infiltration, hypoxia, fibrosis, and metabolic competition, modifies the macrophage response to these radiation-induced signals. At the third level, local mediators such as extracellular vesicle cargo, ROS- and lipid-associated signaling, lactate and NAD+ metabolism, chemokine axes including CCL2/CCR2 and CCL5/CCR5, CSF-1R-dependent recruitment, and concurrent immunotherapy or macrophage-targeting therapy further bias macrophages toward inflammatory M1-like, suppressive M2-like, or hybrid transitional states.

Within that regimen-dependent framework, metabolic and hypoxic signaling appear to function as broad organizing influences because they recur across several tumor models and affect multiple downstream macrophage programs. ROS-lipid signaling, glutamine synthesis, NAD^+^/niacinamide metabolism, lactate production, HIF-1α activity, and hypoxia-associated complement signaling all altered the balance between inflammatory and immunosuppressive macrophage states and influenced the efficacy of radiotherapy alone or in combination with immunotherapy. Likewise, pathways governing macrophage recruitment and persistence, including CCL-family chemokines and CSF-1R signaling, seem to occupy a relatively high functional level because they regulate whether immunosuppressive macrophages accumulate and remain within irradiated tumors. From a therapeutic standpoint, these recruitment and survival pathways may therefore represent some of the more consistently actionable nodes identified in the current preclinical literature.

By contrast, EVs signaling, stromal remodeling, and tumor-intrinsic genomic or transcriptional responses may be better interpreted as relay or context-defining mechanisms rather than universal master regulators. EVs repeatedly transmit biologically important signals between irradiated tumor cells and macrophages, but in the studies their effects are bidirectional; depending on tumor type and vesicle cargo, they can promote either M1-like repolarization and improved antitumor immunity or M2-like polarization, fibrosis, and radioresistance. A similar context dependence is seen with stromal and vascular remodeling, where low-dose vascular normalization may favor inflammatory macrophage states, whereas collagen deposition, stiffness, and pro-metastatic chemokine programs can reinforce M2-associated progression. Tumor-intrinsic genomic and transcriptional responses to radiation also shape macrophage behavior through mediators such as cytokines, growth factors, and ferroptosis-related signals, but the pathways described in this review appear to operate within particular biological settings rather than converging on one single dominant genomic regulator across cancers.

Based on the literature here, a cautious directional prediction can be proposed (**Table 3**). M1-like polarization is more likely when radiotherapy is delivered in immune-activating contexts, such as selected low-dose immune-priming schedules, ablative regimens, ultra-high dose-rate delivery, or particle-based irradiation, particularly when these occur in less hypoxic microenvironments or are combined with treatments that enhance antigen presentation and T-cell activation. In contrast, M2-like or reparative polarization is more likely when irradiation occurs in advanced, hypoxic, lactate-rich, fibrotic, or highly myeloid-infiltrated tumors, especially when the post-irradiation response is dominated by wound-healing signaling, extracellular vesicle–mediated suppression, and sustained monocyte/macrophage recruitment. When these determinants are discordant, mixed or transitional macrophage states should be expected rather than a uniform M1/M2 shift. Accordingly, the most useful interpretation of the current literature is that radiotherapy biases macrophage polarization probabilistically rather than deterministically, and that the dominant direction of polarization can be better anticipated when radiation parameters are interpreted together with tumor ecology and microenvironmental state.

**Table 3:** A working framework for predicting post-radiotherapy macrophage polarization according to radiation regimen, tumor context, and microenvironmental features.

Determinant	Feature/pattern	More likely polarization direction	Representative mechanisms described in this review	Therapeutic implication
Radiation dose and fractionation	Conventional fractionation or prolonged exposure schedules	M2-like or mixed	Tissue-repair signaling, persistent inflammatory stress, chemokine-driven monocyte recruitment, pro-fibrotic remodeling	Consider combining RT with macrophage-recruitment/survival blockade or reprogramming agents
Radiation dose and fractionation	Selected low-dose immune-priming regimens	M1-like or T-cell–permissive mixed states	Increased iNOS-associated programs, vascular normalization, improved immune trafficking in permissive settings	May be useful when the goal is immune priming or enhancing checkpoint responsiveness
Radiation dose and fractionation	Hypofractionated or ablative schedules	M1-like or mixed-to-M1	Stronger immunogenic tumor cell death, enhanced antigen presentation, increased CD8+ T-cell infiltration in some models	May better support combination immunotherapy than conventional fractionation in selected tumors
Radiation modality/delivery	Conventional photons in repair-dominant stromal contexts	Mixed or M2-like	Wound-healing responses, fibrosis, suppressive stromal remodeling	Requires careful contextual interpretation; may benefit from macrophage-targeted combinations
Radiation modality/delivery	Proton therapy	Mixed M1/M2, often shifted away from strongly M2-dominant states	NF-κB activation, altered ROS handling, sustained DNA-damage response in macrophages	May remodel TAMs differently from photons and could be advantageous in rational combinations
Radiation modality/delivery	Carbon-ion radiotherapy	More often M1-like	Stronger antitumor immune profile, reduced immunosuppressive/fibrotic features relative to photons in some models	Potentially favorable platform for combination with immune checkpoint blockade
Radiation modality/delivery	Ultra-high dose-rate/FLASH-type delivery	More often M1-like	Greater shift from M2-like to M1-like states, stronger CD8+ T-cell activation, abscopal immune effects in some models	Supports the idea that dose rate, not only dose, influences macrophage programming
Tumor context	Early-stage, less hypoxic, less fibrotic, less treatment-refractory tumors	More permissive for M1-like polarization	Lower suppressive metabolic burden, less entrenched stromal remodeling, more favorable immune activation	These tumors may be more responsive to RT-driven immune priming
Tumor context	Advanced, recurrent, hypoxic, fibrotic, or treatment-resistant tumors	More permissive for M2-like polarization	Chronic inflammatory signaling, matrix remodeling, angiogenesis, immune escape programs	Stronger rationale for combining RT with TAM-depleting or TAM-reprogramming strategies
Tissue/myeloid ontogeny context	Organ-specific resident macrophage compartments and distinct stromal niches	Context dependent; often mixed	Differences in macrophage origin, transcriptional responsiveness, vascular architecture, ECM, and hypoxia across organs	Helps explain why similar RT regimens may produce different TAM states in brain, liver, pancreas, lung, breast, or rectum
Metabolic and hypoxic context	Lower lactate burden/weaker hypoxia-associated suppression	More permissive for M1-like states	Reduced HIF-1α/lactate-driven immunosuppression; less dominance of reparative metabolic signaling	Metabolic normalization may improve immune activation after RT
Metabolic and hypoxic context	Lactate-rich, hypoxic, oxidized lipid–rich, NAD+/niacinamide- or glutamine-reprogrammed environments	M2-like	HIF-1α signaling, lactate accumulation, ROS-lipid/PPARγ pathways, glutamine synthetase activity, EV-mediated metabolic suppression	Supports combining RT with metabolic reprogramming approaches
Recruitment and survival pathways	High CCL2/CCR2, CCL5/CCR5, CCL7/CCL8, or CSF-1R activity after irradiation	M2-like accumulation/persistence	Recruitment of circulating monocytes, maintenance of immunosuppressive TAMs, enhanced post-RT tumor support	Strong rationale for chemokine-axis blockade or CSF-1R inhibition
Recruitment and survival pathways	Pharmacologic blockade of CCL-family axes or CSF-1R	Shift toward M1-like/reduced M2 burden	Reduced TAM recruitment, reduced survival of protumor macrophages, improved antitumor immunity	Among the more consistently actionable macrophage-targeting strategies in the review
Extracellular vesicle signaling	EV cargo from irradiated or aggressive tumors that carries suppressive metabolic/miRNA signals	M2-like	Tumor-to-macrophage transfer of signals that favor immune suppression, fibrosis, or radioresistance	Suggests value of targeting tumor-derived EV pathways in selected settings
Extracellular vesicle signaling	RT-associated microparticles or EV cargo that promotes immunogenic stress and phagocytosis	M1-like	Enhanced macrophage phagocytosis, ferroptotic/immunogenic signaling, improved CD8+ T-cell crosstalk	Can be exploited for radio-immunotherapy design
Combination therapy	RT combined with checkpoint blockade, CD40 agonists, PI3Kγ inhibition, STING/TLR agonists, selected anti-angiogenic or CAR-T strategies	More likely M1-like repolarization	Enhanced antigen presentation, reduced suppressive signaling, increased CD8+ T-cell recruitment and function	The direction of macrophage polarization becomes more favorable when suppressive pathways are blocked or immune activation is amplified
Combination therapy	RT combined with therapies that reinforce suppressive remodeling or blunt immune activation	Mixed or M2-like persistence	Failure to sustain cytotoxic immunity, persistence of suppressive myeloid programs	Combination selection matters; not all RT-based regimens promote favorable macrophage remodeling
Overall interpretive rule	Determinants are discordant across regimen, tumor type, and microenvironment	Hybrid/transitional states expected	Coexistence of inflammatory, reparative, and immunosuppressive cues	Supports using “M1-like” and “M2-like” as functional shorthand, not rigid endpoints

EV, extracellular vesicle; RT, radiotherapy; TAM, tumor-associated macrophage. This framework is intended as a working, hypothesis-generating synthesis of the literature reviewed here rather than a validated clinical prediction model. In this review, post-radiotherapy macrophage polarization is interpreted as probabilistic and context dependent, with mixed or transitional states expected when treatment-related and microenvironmental determinants are discordant.

This hierarchy also helps clarify which mechanisms may matter most in different clinical scenarios. In hypoxic or metabolically rewired tumors, metabolic and hypoxia-associated pathways are likely to be especially relevant. In highly myeloid-rich tumors or settings marked by strong post-irradiation monocyte influx, recruitment and survival pathways may be more decisive. In contrast, when the goal is to enhance systemic immunity or generate abscopal responses, downstream processes such as antigen presentation, phagocytosis, EV-mediated signaling, and macrophage-T-cell coordination appear particularly important. Finally, in radiation-induced normal tissue injury, especially at mucosal sites, microbiome-associated macrophage programming seems more relevant to radioprotection than to tumor radiosensitization. Because most evidence reviewed here remains preclinical and tumor-specific, this proposed hierarchy should be viewed as a working framework rather than a definitive ranking.

## 10 Conclusion

Radiotherapy reshapes the TME in ways far more intricate than its classical definition as a DNA-damaging modality implies. Across tumor types, models, and radiation platforms, a central theme emerges: macrophage polarization is both a consequence of irradiation and a determinant of therapeutic outcome. Ionizing radiation initiates a cascade of metabolic, genomic, and microenvironmental adaptations—ranging from ROS and lipid peroxidation to cGAS–STING activation, hypoxia-driven complement signaling, and tumor-derived extracellular vesicle remodeling—that converge on the myeloid compartment. The resulting macrophage phenotypes can either amplify antitumor immunity through M1-associated inflammatory programs or reinforce repair, angiogenesis, and immune suppression through M2-skewed states. These diverging trajectories help explain why radiotherapy may induce systemic tumor control in some contexts while promoting recurrence, metastasis, or stromal fortification in others. The apparently divergent roles of ROS across studies reflect differences in timing, dose rate, intracellular localization, and downstream signaling: acute ROS bursts linked to immunogenic cell death may support M1 polarization and tumor control, whereas sustained oxidative stress or ROS-driven lipid/metabolic programs can instead reinforce M2-like, pro-tumor macrophage states.

Radiation modality and dose further refine these macrophage responses. High-dose ablative photons, ultra-high dose-rate delivery, and particle-based approaches such as protons and carbon ions more consistently promote inflammatory M1-like signatures and enhance synergy with checkpoint blockade or CAR-T cell therapy. Conventional fractionation, hypoxia, and radiation-induced stromal activation more often support M2-associated resistance programs. These shifts are neither uniform nor unidirectional; instead, they reflect a dynamic balance between immunogenic cell death signals and pro-repair cues released from irradiated tumor, stromal, and endothelial compartments.

Therapeutically, this complexity is increasingly actionable. Strategies that deplete, reprogram, or restrict the recruitment of TAMs—through CSF-1R blockade, CCR2/CCR5 inhibition, PI3Kγ targeting, MerTK suppression, or CD^40^ agonism—can resensitize tumors to radiotherapy and expand the window for durable immune control. Nanoparticle-based radiosensitizers, metabolic modulators, engineered bacteria, and immunostimulants such as STING or TLR agonists further leverage the plasticity of macrophages, converting radiation-exposed tumors to *in situ* vaccines capable of supporting abscopal responses. At the same time, interventions that protect normal tissues—from microbiome stabilizers to targeted radioprotectants—operate partly by restoring anti-inflammatory M2-like programs in mucosal environments, illustrating that macrophage polarization underlies not only therapeutic efficacy but also treatment-related toxicity.

From a translational standpoint, macrophage-focused biomarkers including exosomal cargo, interferon-associated signatures, radiosensitivity indices, and tissue-level macrophage states are emerging as potential tools for patient stratification, while imaging and peripheral immune monitoring may help track treatment-induced myeloid remodeling in real time. However, clinical implementation will depend not only on biomarker discovery, but also on overcoming major practical barriers, including drug delivery into spatially heterogeneous tumor niches, insufficient specificity of current TAM-targeting approaches, the possibility of systemic immune or normal-tissue toxicity, and the limited fidelity of murine models for human tumor immunology. Progress in this field will therefore require biomarker-guided, spatially informed, and temporally optimized strategies rather than uniform macrophage depletion or oversimplified repolarization approaches.

Collectively, the evidence positions macrophages not as passive bystanders but as central mediators of radiotherapy’s duality—capable of enabling tumor regression or fostering resistance depending on how they are shaped by radiation-induced signals. Future progress will likely depend on integrating macrophage-targeted interventions with optimized radiation parameters and immunotherapies, tailoring strategies to the metabolic, stromal, and inflammatory context of each tumor type. As radiotherapy continues to evolve into a platform for immune modulation rather than solely cytotoxic injury, understanding and directing macrophage polarization will be key to improving both local control and systemic outcomes, and to narrowing the gap between preclinical promise and clinical implementation.
